# Decline of coastal apex shark populations over the past half century

**DOI:** 10.1038/s42003-018-0233-1

**Published:** 2018-12-13

**Authors:** George Roff, Christopher J. Brown, Mark A. Priest, Peter J. Mumby

**Affiliations:** 1 School of Biological Sciences, University of Queensland, Brisbane, QLD 4072 Australia; 2 Australian Rivers Institute, Griffith University, Nathan, QLD 4111 Australia

**Keywords:** Population dynamics, Conservation biology, Ecology, Community ecology

## Abstract

Overexploitation of large apex marine predators is widespread in the world’s oceans, yet the timing and extent of declines are poorly understood. Here we reconstruct a unique fisheries-independent dataset from a shark control programme spanning 1760 km of the Australian coastline over the past 55 years. We report substantial declines (74–92%) of catch per unit effort of hammerhead (Sphyrnidae), whaler (Carcharhinidae), tiger shark (*Galeocerdo cuvier*) and white sharks (*Carcharodon carcharias*). Following onset of the program in the 1960s, catch rates in new installations in subsequent decades occurred at a substantially lower rate, indicating regional depletion of shark populations over the past half a century. Concurrent declines in body size and the probability of encountering mature individuals suggests that apex shark populations are more vulnerable to exploitation than previously thought. Ongoing declines and lack of recovery of vulnerable and protected shark species are a cause for concern.

## Abstract (web summary)

George Roff et al. report a 74–92% decline in the catch per unit effort and concurrent declines in body size of large coastal apex sharks near Australia over the past five decades. This study highlights shifting baselines of shark populations in coastal shark assemblages, and indicates that shark populations are highly vulnerable to exploitation.

## Introduction

Through hunting and widespread habitat modification, top-level predators have been depleted throughout the world’s continents, rivers and oceans^1^, driving widespread ecological change^2^. While losses of apex predators and the consequences for ecosystems have been well documented in terrestrial ecosystems^1^, the extent and magnitude of decline in apex predators in the marine environment is less well understood^3,4^. Coastal ecosystems in particular have experienced widespread trophic downgrading, having lost many of their top-level predators through overfishing^5^. Throughout the 20th century, increasing human-shark interactions in coastal ecosystems lead to the public perception that sharks are dangerous to people, resulting in near extirpation of some coastal shark species through hunting^6^. Despite widespread evidence for historical exploitation of coastal sharks, historical baselines for population sizes are largely unknown^7^. The absence of baselines is particularly problematic for conservation of endangered and threatened shark populations, and the extent to which targeting shark populations reduces interaction rates with humans in coastal ecosystems is contentious^8^.

**Fig. 1:**
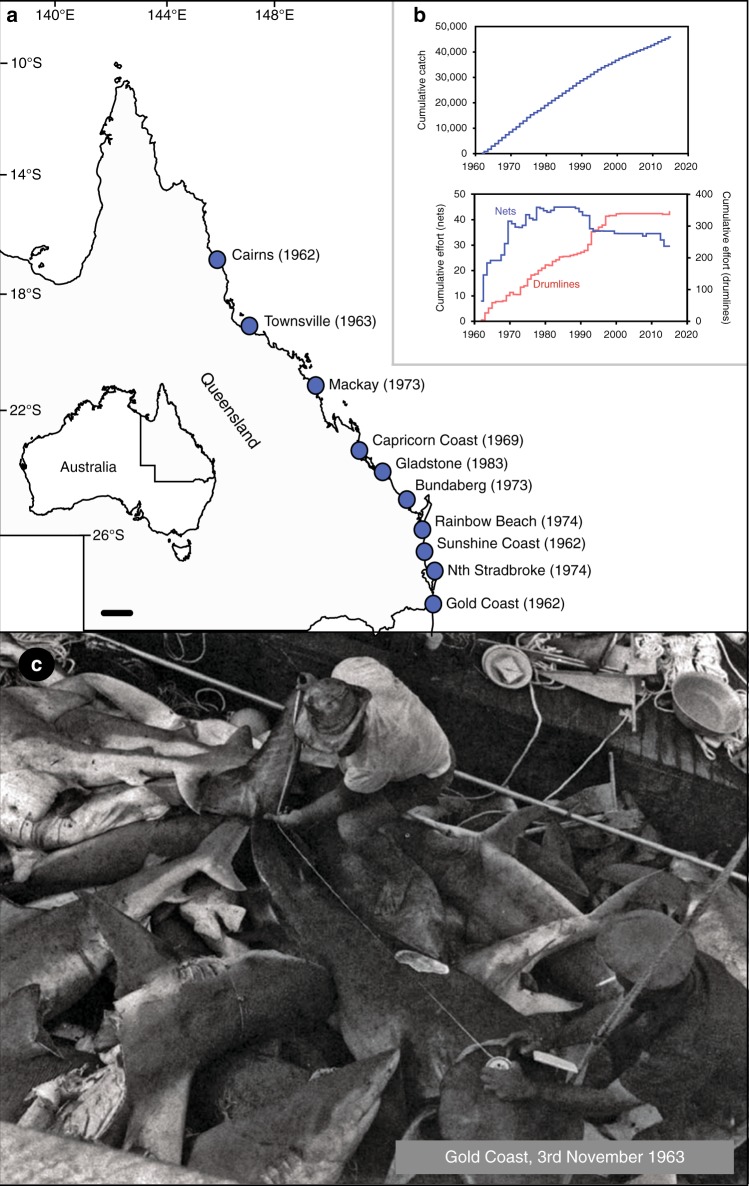
**Regional setting and historical changes in catch and effort for the Queensland Shark Control Program. a** Timing of the establishment of shark control programmes across the Queensland coastline (map created under Creative Commons Attribution 4.0 International from Geoscience Australia). **b** Cumulative effort for nets and drumlines and catch between 1962 and 2017. **c** Historical photograph of contractors measuring sharks removed from QSCP nets on the Gold Coast in the early years of the programme (3 November 1963), reprinted from Paterson (1990) Biological Conservation, 52(2), 147–159 (ref. ^17^) with permission from Elsevier

Here we report on long-term changes in shark catches from the Queensland Shark Control Program (QSCP) adjacent to the Great Barrier Reef World Heritage Area (GBRWHA). The QSCP has been operating since 1962 using a system of mesh nets and baited drumlines (Supplementary Figure 1) with an aim to “minimise the threat of shark attack on humans”^9^ by reducing the local populations of large sharks to minimise the probability of encounters between sharks and swimmers^10^. The programme started in Cairns in 1962, and has since expanded to 11 regions in Queensland, spanning tropical and sub-tropical coastal ecosystems across 1760 km of the eastern Australian coastline^11^ (Fig. 1a, Supplementary Figure 2). To date, nearly 50,000 sharks have been caught by the QSCP (Fig. 1). From the onset of the programme in 1962, increasing numbers of baited drumlines were installed in place of nets (Fig. 1b) due to logistical constraints and issues of bycatch (predominantly turtles and dugongs^11^).

## Results

### Species identification and taxonomic composition of shark catch

Analysis of the QSCP catch data reveals a diverse range of sharks (45 spp.) spanning multiple trophic levels (Supplementary Table 1), ranging from small (~ 80 cm maximum total length [TL_max_]) low trophic level sharks (e.g. *Heterodontus portusjacksoni*) to large (> 600 cm TL_max_) apex sharks such as tiger sharks (*Galeocerdo cuvier*) and white sharks (*Carcharodon carcharias*). Although the QSCP has been in operation since 1962 (Fig. 1c), records relating to species identification are considered reliable only from ~1996 onwards following a systematic review of the programme^9^. Shark catches from the long-term data set (1962–2017) were therefore grouped into five broad categories based on reliably identifiable characteristics: (1) hammerhead sharks (Sphyrnidae, 23% of total catch, predominantly *Sphyrna mokarran* & *Sphyrna lewini)*, (2) tiger sharks (*Galeocerdo cuvier*, 26% of total catch), (3) whaler sharks (requiem sharks of the family Carcharhinidae, 45% of total catch), (4) white sharks (*Carcharodon carcharias*, 2% of total catch) and (5) other sharks (4% of total catch, Supplementary Table 1).

### Long-term changes in CPUE of shark populations

**Fig. 2:**
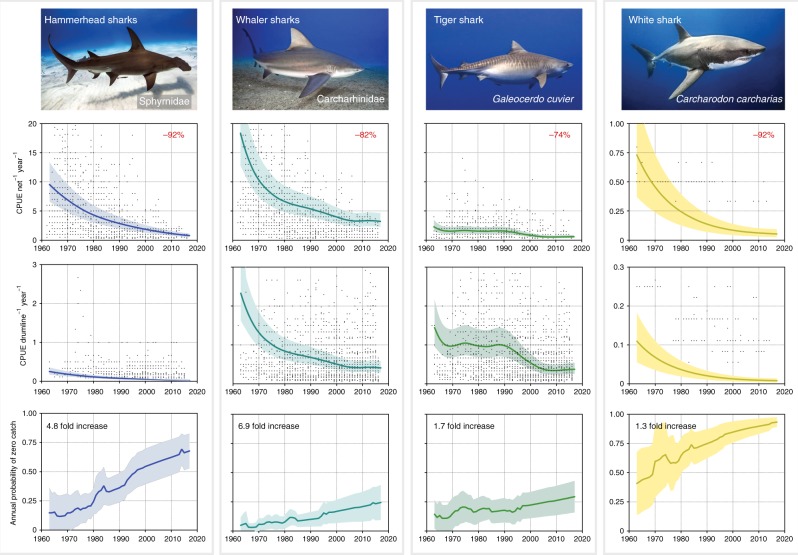
Catch per unit effort (CPUE) in nets and drumlines with fits from Bayesian negative binomial generalised additive mixed effects models (± 95% credibility intervals), and the annual zero-catch probability (± 95% credibility intervals). Percentages represent the % decline over the 1962–2017 dataset. Photographs courtesy of Juan Oliphant (http://oneoceandiving.com/)

Bayesian negative binomial mixed effects models revealed substantial declines in catch per unit effort (CPUE) of large apex sharks over the past five decades (Supplementary Table 2). In 1962, an average of 9.5 hammerheads were recorded per net per year, which declined by 92% to 0.8 hammerheads in 2016 (Fig. 2a). These declines did not appear to follow a latitudinal gradient and were consistent among regions (Supplementary Figure 3). Hammerhead sharks are more vulnerable to capture in nets owing to their unique hammer shaped cephalofoil that easily becomes entangled^11,12^ (Supplementary Figure 3). Drumline catch declined from an average of 0.25 hammerheads per drum per year in 1962 to 0.02 in 2016 (Fig. 2a). Whaler sharks (Carcharinidae) also exhibited large declines in CPUE: in 1962, catches averaged 18.3 individuals per net per year declining by 82% to 3.23 individuals per net per year by 2016, while catch rates of drumlines declined from 2.3 individuals per drum per year in 1962 to 0.4 in 2016 (Fig. 2b). Declines in hammerheads and whalers were exceptionally rapid following the deployment of nets in the early 1960s (Fig. 1b), exceeding an exponential rate of decline (Fig. 2a, b). By the mid 1970s, average hammerhead catch rates were 45–55% lower than the previous decade, and CPUE continued to decline, reaching ~ 75% of historical baselines by the mid 1990s. Coinciding with decreasing catch rates, the estimated annual zero-catch probability (catching no hammerhead sharks at any given beach within a region per year) increased by 4.8-fold between 1962 and 2016 (Fig. 2a), while the annual zero-catch probability of whalers increased by 6.9-fold in the same time-period (Fig. 2b). These trends were broadly consistent across all regions (Supplementary Figure 4). As whaler sharks encompass a broad group of sharks within the Carcharinidae family (26 spp., Supplementary Table 1), the lack of species-resolution in the long-term dataset renders it unclear as to whether the 1962–2016 decline in catch rates represents an even decline among whalers, or masks long-term shifts in species composition among members of the Carcharinidae.

In contrast to hammerheads and whalers, catch rates of tiger sharks were relatively stable between the early 1960s and early 1990s, prior to a 74% decline in CPUE over the past 25 years (Fig. 2b). Catches of 1.4 individuals per drum per year in 1962 declined to 0.4 individuals in 2016, while catches in nets declined from 2.3 individuals per net per year in 1962 to 0.6 individuals in 2016 (Fig. 2c). Coinciding with ongoing declines in numbers of tiger sharks in nets and drumlines, the annual zero-catch probability of tiger sharks increased by 1.7-fold (Fig. 2c). The strongest declines in tiger shark CPUE were recorded at high latitude regions (Supplementary Figure 5), where tiger sharks undergo seasonal migrations in warmer summer months^13^. Such a result is broadly consistent with previous observations of geographic range constriction commensurate with population declines in pelagic predators^14^.

While relatively uncommon (~ 2% of total catch), white sharks are considered ‘high risk’ and are actively targeted by the QSCP^11^, despite being listed as ‘vulnerable’ under the Australian Environment Protection and Biodiversity Conservation Act (EPBC) in 1999. CPUE of white sharks in the QSCP declined by 92% over the past five decades from 0.7 white sharks per net per year in 1962 to 0.05 individuals in 2015, and 0.1 white sharks per drum per year in 1962 to 0.008 individuals in 2015 (Fig. 2d). These declines are ongoing in 8 out of 9 regions (Supplementary Figure 5) despite a complete ban of commercial and recreational fishing of white sharks since 1999 under the environment protection and biodiversity conservation act and the enactment of a 2002 recovery plan by the federal government^15^.

As is common when reconstructing historical baselines^16^, some degree of uncertainty exists in the accuracy of effort records in the early years of the QSCP. Prior to the review and standardisation of the programme in 1992, exact setting of nets may have varied among regions, and differences in hook types and bait on the drumlines may have occurred among regions and through time. Similarly, the accuracy of catch records may be questioned as historical data has been collected by contracted commercial fisherman prior to standardised training in shark species identification from 1992 onwards. While minor gear variation may have occurred, declines in CPUE were spatially consistent among regions in the early years of the programme along nearly 1800 km of coastline (Fig. 1), and analysis of temporal trends in bycatch revealed no clear evidence of changes in gear types in the early years of the programme coinciding with rapid declines in shark catches^17–20^. Further, it is unlikely that changes in drumline gear and bait types would have a substantial effect on CPUE as large sharks are omnivorous and opportunistic^12,21,22^, and likely do not exhibit strong preference for fish or shark flesh as bait. In addition, changes in hook types during by-catch reduction trials have no measurable effect on shark catches^23^, and declines were consistent in both nets and drumlines (Supplementary Figure 3). Notes from contractors during the early years of the programme provide insight into changing shark dynamics and support the observed decline in CPUE: in some regions nets were installed shortly after the programme initiated to cope with “increase(s) in large shark catches” (contractor notes), while the number of drumlines in some regions were reduced in the 1990s due to declining catch rates of sharks. Patterson^17^ notes that “When net catches were high in earlier years, reliance on lines was unnecessary to achieve satisfactory results but as net catches declined lines were used more assiduously in some regions, with a consequent increase in tiger shark catches”.

While some uncertainty exists with the historical data, analysis of the detailed contemporary catch data since standardisation of nets and drumlines throughout the region and following formal training of contractors in shark species identification (1992–2017) reveals ongoing declines over the past 25 years (Supplementary Figure 6). CPUE of hammerheads declined by 68%, whalers by 69%, tigers by 69% and white sharks by 42%, while the probability of catching no sharks at any given beach within a region increased through time (Supplementary Figure 6). While ongoing declines are a cause for concern, historical data from the long-term dataset (1962–2017) suggest that the historical baselines for populations may be substantially higher than that based on contemporary data. This represents a classic case of shifting baseline syndrome^5,24^, and implies that studies of sharks declines in the region in recent decades^12,25,26^ may be predicated on a substantially shifted baseline.

### Regional depletions of shark populations

**Fig. 3:**
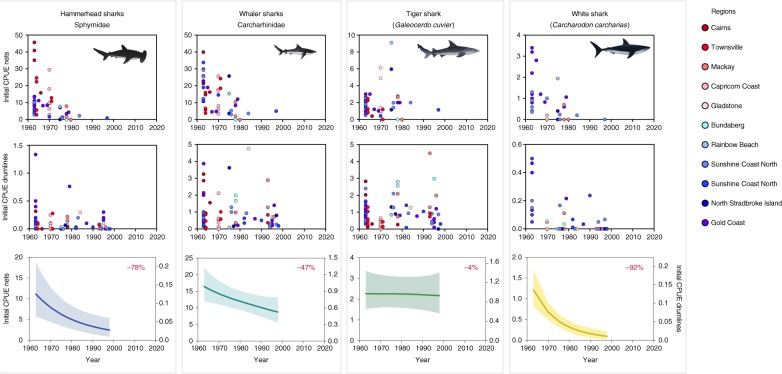
Initial catch per unit effort (defined as the average CPUE of the first five years of operation) for each beach within regions, and model fits from Bayesian generalised additive mixed effects models (± 95% credibility intervals) for nets and drumlines. Symbols courtesy of the Integration and Application Network (ian.umces.edu/symbols/)

Shark control programs operate with the intent of depleting local populations of sharks, yet the spatial scale at which these depletions occur is not well understood. Following the initial deployment of shark nets in Cairns and the Gold Coast in 1962 (Fig. 1), the programme expanded along the Queensland coastline to include additional beaches and additional regions between 1962 and 1998 (Supplementary Figure 2). To quantify the spatial scale of population declines, we explored changes in initial catch rates (calculated as the average CPUE for the first five years) following the installation of gear at new beaches. Within regions, CPUE varied substantially among beaches within years, consistent with differential habitat preferences and environmental drivers of shark distributions that operate over relatively small spatial scales^27,28^. Despite such small-scale variability in CPUE, initial catch rates in newly installed beaches were consistently similar to that of established beaches within regions (Supplementary Figure 7). At regional scales, initial CPUE in new net and drumline installations in recent decades occurred at a lower rate than earlier installations for hammerhead, whaler and white sharks (Fig. 3). Analysis of this trend using Bayesian mixed effects models indicates a decline in initial CPUE between 1963 and 1998 of 78% for hammerheads, 47% for whalers and 92% for white sharks (Fig. 3). Decline in initial catch rate was considerably smaller for tiger sharks (−4%, Fig. 3), which is consistent with relatively stable CPUE of tiger sharks between 1960 and ~1990 prior to region-wide declines CPUE in the late 1990s (Supplementary Figure 6).

From a management perspective, assessing the status of stocks through fisheries data can be problematic, as CPUE may be decoupled from abundance due to a range of behavioural and operational factors that can affect catch rates^29^. The initial declines in CPUE have been theorised to reflect depletions of local populations, with subsequent catches comprising an influx of sharks from adjacent regions^30^. Such a response would result in hyperdepletion, a phenomena by which CPUE declines more rapidly than population abundance^31^. The impact of shark control programs upon populations will vary among species, and is likely dependant on both movement patterns, habitat use and the degree of philopatry^32^. In theory, hyperdepletion would be more likely to occur in whaler sharks that exhibit small-scale movements and site attachment within bays on the Queensland coastline^33^ than larger apex species that undergo large-scale transoceanic migrations^34^ and whose populations cover entire ocean basins^35^. Nevertheless, the advent of satellite tracking of sharks presents an emerging picture that even apex species that undergo long distance movements (>1000 km), including tiger^36^, hammerhead^37^ and white sharks^38,39^, can exhibit patterns of residency or site-attachment (see ref. ^40^ for a concise review), rendering them susceptible to localised depletion in shark control programs. Indeed, declines in the early years of the programme and increases in the probability of annual zero catches for these taxa may represent selective depletion of site attached or resident individuals from the regional population. However, the aseasonal migration of sharks to coastal nursery areas adjacent to the QSCP^12,39^ would favour patterns of hyperstability (e.g. ref. ^41^.) rather than hyperdepletion. The ongoing reduction in initial CPUE as the programme expanded implies that the scale of declines extend beyond local beaches where the shark control programme operates, and points to serial depletion of large apex sharks throughout the wider region over the past five decades. Dynamics population models should now be developed to explore the causes of declines and policy options for reversing them (e.g. refs. ^42,43^).

### Long-term changes in size structure

Life-history characteristics, such as growth, longevity and fecundity are largely correlated with body size in sharks^44,45^. Size is also strongly linked to trophic position^4,45^, and size-structuring in communities can be a strong determinant of the strength of competitive and predatory interactions^45^. Coinciding with substantial declines in CPUE over the long-term dataset (1962–2015), the average size of hammerhead sharks (Sphyrnidae) increased over the past five decades by 5% (210–221 cm, Supplementary Table 3). As ‘Sphyrnidae’ encompasses both the large great hammerhead (*Sphyrna mokkaran*, TL_max_ = 610 cm^46^) and the smaller scalloped hammerhead (*Sphyrna lewini*, TL_max_ = 430 cm^46^), it is unclear whether the increase in size through time reflects a shift in the proportion of scalloped vs great hammerheads, or alternatively reflects selective declines of neonate and juvenile scalloped hammerhead sharks from adjacent coastal nursery grounds^12^ in the early years of the programme. For the past two decades where species-specific data are available (1997–2017), the average size of great hammerheads declined significantly by 22% (274–215 cm) and scalloped hammerheads by 16% (204–177 cm, Supplementary Table 4).

**Fig. 4:**
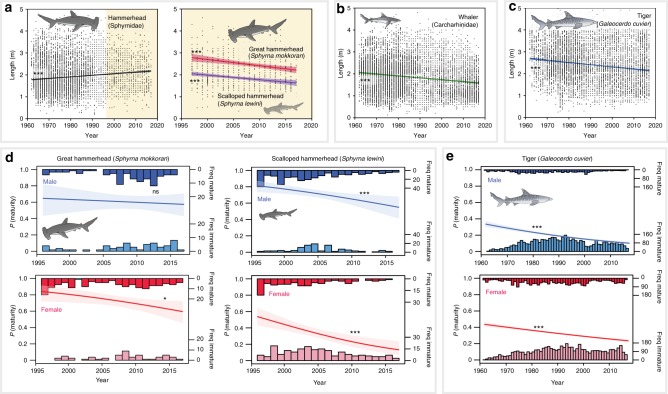
Long-term changes in size structure and sexual maturity. Linear regressions (± 95% confidence intervals) for change in size for **a** hammerheads (1962–2017, shading indicates period in which species specific data are available), great and scalloped hammerheads (*Sphyrna mokarran* and *Sphyrna lewini*, 1997–2017), **b** whaler sharks (Carcharhinidae, 1962–2015), **c** tiger sharks (*Galeocerdo cuvier*, 1962–2017), and binomial probability models for sexual maturity in male and female, **d** great and scalloped hammerheads (*S. mokarran* and *S. lewini*, 1997–2017) and **e** tiger sharks (*G. cuvier*, 1962–2017). ns not significant. ***p < 0.001, **p < 0.01, *p < 0.05. Symbols courtesy of the Integration and Application Network (ian.umces.edu/symbols/)

The average size of tiger sharks declined significantly by 21% (272–215 cm) over the past five decades, a pattern that was consistent among males and females (Supplementary Figure 8). The average size of whalers also declined significantly by 9% between 1962 and 2017 (193–166 cm). Long-term declines in the whaler group may reflect an overall intraspecific reduction in size over the past five decades, and/or shifts in species composition towards smaller species of whalers. While our results provide insight into long-term changes in size structure of large apex shark populations, a degree of uncertainty exists in historical records in the early years of the programme. In the early years prior to 1990, a bounty system was in place for large sharks over two metres in size, which may have provided an incentive to exaggerate the sizes of smaller sharks by contractors for monetary gain. While the extent to which this occurred is unclear, significant declines in size among hammerhead, whaler and tiger sharks have continued over the past 20 years following the removal of bounties and review of the QSCP (1997–2017), and the rate of decline in the size of tiger sharks across the long-term dataset 55-year dataset (10.4 cm per decade, Fig. 4), is similar to that occurring over the past 20 years (8.6 cm per decade).

From a demographic perspective, species with large maximum sizes, low growth coefficients, low fecundity, and higher size at maturity specifically targeted by the QSCP (Supplementary Figure 9) are particularly vulnerable to overfishing^44^. We used previously published estimates of sizes at maturity of shark populations from local studies^27,47,48^ to quantify changes in the probability of maturity in hammerhead and tiger shark catches in the QSCP over the past two decades. While our estimates assume that size at maturity is fixed over time, sharks are unlikely to exhibit rapid shifts in maturity due to their *K*-selected life-history strategies^44^. Our results indicate significant and substantial declines in the probability of recording mature male and female scalloped hammerheads over the past 20 years (Fig. 3d). Most notably, the probability of recording mature females of scalloped hammerheads declined from 54% in 1997 to 14% in 2017, while probability of mature males declined from 82 to 55% over the same time-period (Supplementary Table 5). Significant declines were also recorded for female great hammerheads (85% in 1997 to 59% in 2017, Fig. 3d), although no declines were observed for male great hammerheads. Over the past 50 years, the probability of recording sexually mature female tiger sharks declined from 43% in 1962 to 23% in 2017, while the probability of recording mature males declined from 34 to 9%. In contrast to other sharks caught by the QSCP, most white sharks were juveniles and sub-adults, and very few (<1% of females and 7% males) were of mature size. Considering that the QSCP selectively targets larger sharks (> 1.5 m^11^), the ongoing decline in the probability of catching mature hammerheads and tiger sharks is of concern, as declines in the number of sharks reaching maturity can strongly influence population dynamics and inhibit recovery rates^44^.

### Causes of declines in coastal shark populations

**Fig. 5:**
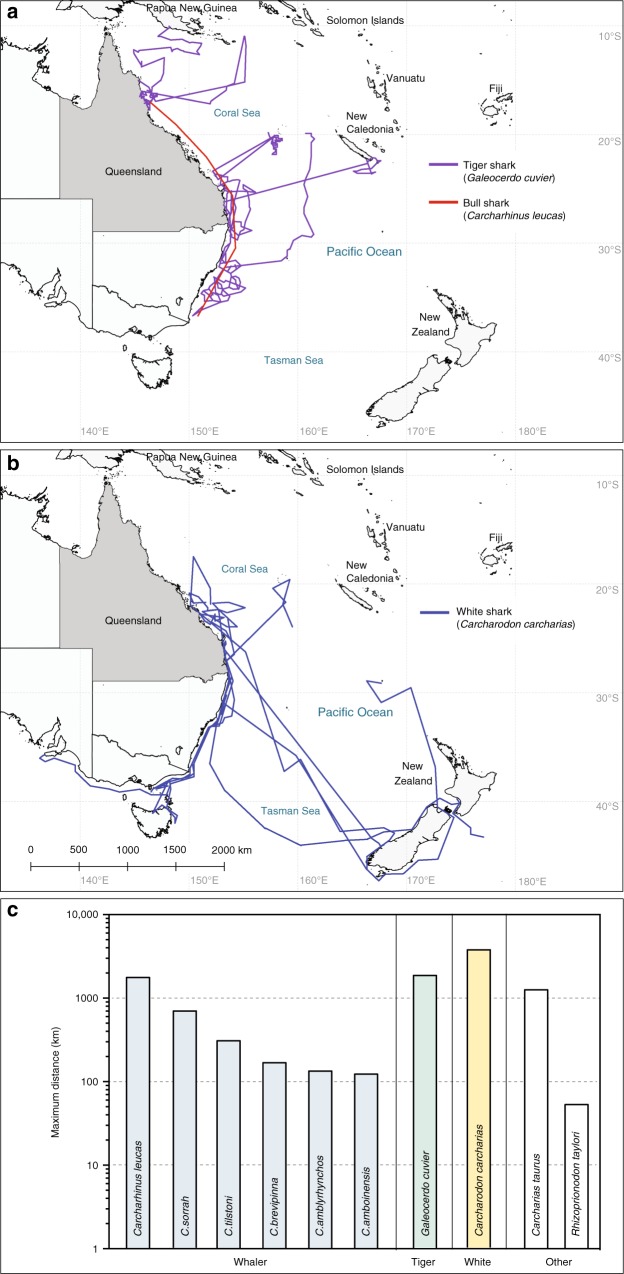
Regional movement patterns among coastal and oceanic ecosystems. Representative movement tracks derived from satellite and acoustic tracking studies of sharks adjacent to the eastern Australian coastline for **a** tiger sharks (*Galeocerdo cuvier*, *n* *=* 10) and bull sharks (*Carcharhinus leucas*, *n* = 17), **b** white sharks (*Carcharodon carcharias*, *n* = 6), and **c** maximum distance derived from movement studies for whaler sharks (Carcharhinidae), tiger sharks (*G. cuvier*), and white sharks (*C. carcharias*) caught in the QSCP program

With widespread depletions of sharks throughout the world’s oceans, conservation and management of shark populations is becoming increasingly important^49^. Given the multi-jurisdictional nature of apex shark movement^50^ and paucity of historical records, causes of declines at regional scales can often be hard to pinpoint, and vary substantially among species. The rate and magnitude of declines in CPUE across multiple taxa strongly implicate fishing as the primary cause of long-term declines, and precludes alternative hypothesis such as environmental drivers or shifting prey dynamics (Supplementary Table 6). To assess the potential role of fisheries in the timing of decline of coastal shark populations, we compiled available records of local and regional commercial and recreational fisheries from the mid-20th century (Supplementary Table 7). As nets and baited drumlines are highly efficient in catching sharks, the QSCP is likely to have exerted localised impacts on coastal shark populations in regions where gear has been deployed. Indeed, shark control programmes are considered effective because they systematically target and reduce populations of large sharks that are believed to be dangerous^9^. At regional scales, considering the widespread movement patterns of large apex sharks (Fig. 5) and genetic evidence for population connectivity among Australian waters and throughout the Indo-Pacific^35,39,51,52^, the serial declines in shark populations recorded by the QSCP likely also reflects ongoing population depletion by recreational, and commercial fisheries in Queensland and adjacent jurisdictions, although the absence of historical fisheries data from the 1960s and 1970s makes the early causes of declines difficult to pinpoint. The rapid initial declines indicate that apex sharks may be susceptible to even relatively low levels of fishing pressure^3,44^. While the annual regional catch of whalers and hammerheads in Queensland net fisheries in recent years exceeds the annual catch in the QSCP by nearly an order of magnitude (Supplementary Figure 10), the targeting of both neonate and juvenile life stages and selective removal of large individuals in recreational and commercial fisheries (Supplementary Table 7) coinciding with the expansion of shark control programs in QLD and adjacent New South Wales^53^ is likely to have had a substantial impact on sharks with low population growth rates.

As tiger sharks are not generally considered a target species by fisheries within the region (Supplementary Table 7), the initial stability in CPUE may reflect either a lack of fishing pressure in the early years of the programme, or alternatively may reflect shifts in the species composition of shark assemblages in response to overall population declines^54^. The ongoing declines in CPUE, increase in probability of zero catch and reduction in size of tiger sharks that started in the 1990s coincides with a near doubling in the number of baited drumlines in 8 of the 11 regions (Supplementary Figure 2). Additional increases in recreational and commercial fisheries for tiger sharks over the past 20 years indicates that current fishing pressure of these sharks may be unsustainable^25^. As a consequence of local and regional exploitation, large apex sharks that were once historically abundant are now considered ‘Endangered’ and ‘Vulnerable’ under the IUCN Red Listing (Supplementary Table 1). Critically, white sharks are now increasingly rare in QSCP catch, having undergone a 92% decline over the past five decades. The apparent lack of recovery of protected white shark populations despite a complete ban on commercial and recreational fishing in Queensland and neighbouring New South Wales over two decades ago^15^ is a cause for concern, and implicates ongoing catches in shark control programs on the eastern Australian coastline and fisheries bycatch as drivers of population declines.

### Regional movements and connectivity of shark populations

As top-level consumers, apex sharks exhibit widespread movements throughout the world’s oceans^4,40,50^. Thus, population declines in coastal habitats may have cascading effects in adjacent coastal and pelagic ecosystems. To quantify the scale of shark movements and potential for connectivity among coastal habitats, we synthesised biotelemetry and tagging data from previous studies along Queensland coastline (*n* = 436 sharks, Supplementary Table 8). Movement patterns of great and scalloped hammerhead shark species on the Queensland coastline are currently unknown. Genetic evidence supports connectivity of scalloped hammerheads along the continental shelf between Australia and Indonesia^52^, and although speculative, analysis of population structure suggests that adult females may migrate from Australia to Indonesia and Papua New Guinea^55^. Evidence from tagging studies in the Atlantic indicate that while great hammerheads undergo large-scale (> 3000 km) oceanic migrations, they also exhibit seasonal residency to coastal and coral reef ecosystems and long-term site fidelity^37^. Most species of whaler sharks for which data are available exhibited varying patterns of residency, dispersal and connectivity among coastal environments on the Queensland coastline (Fig. 5), long-range migrations and multiple habitat use among coastal and coral reef ecosystems was observed in bull sharks (*Carcharhinus leucas*^56^).

Satellite tracking data from tiger sharks on the Queensland coast indicate smaller scale resident behaviour^36^ coupled with widespread movement along the eastern Australian coastline (23°S–40°S, Supplementary Table 8). Tiger sharks have been reported to migrate to higher latitudes in warmer months for foraging^36^, and tracking studies provide evidence of long-distance migrations (>1000 km) from the Queensland coast to tropical coral reef regions of New Caledonia and Papua New Guinea (Fig. 5). Such widespread movement patterns are consistent with recent studies indicating population connectivity spanning among eastern and western Australia, and Hawaii, resulting in a single large Indo-Pacific population of tiger sharks^35,51^. Tracking data for white sharks indicate that while movements are predominantly linked to nearshore waters along the eastern Australian coastline (23°S–39°S, Supplementary Table 8), large-scale (> 3000 km) transoceanic excursions were recorded from subtropical Queensland to temperate New Zealand waters (Fig. 5). Such widespread movement patterns of large apex sharks among coastal and pelagic ecosystems indicates a degree of connectivity among habitats (sandy beaches, coral reefs, seagrass beds, kelp forests) along the eastern coastline of Australia and throughout Oceania (Fig. 5). Depletion of shark populations recorded on the Queensland coastline over the past 50 years may have had cascading effects on broad-scale nutrient transfer and cross-ecosystem linkages among adjacent food webs throughout the region^57,58^.

## Discussion

In terrestrial ecosystems, habitat loss and hunting have been the primary drivers of decline in large vertebrate species^1,2^. The removal of large carnivores in terrestrial systems has substantial impacts at ecosystem scales^1,2^, which is often at direct odds with conservation objectives^59^. Hunting to reduce conflict is prevalent in terrestrial ecosystems, yet the extent to which it occurs in marine ecosystems is largely undocumented. While the efficacy of shark control programs remains controversial, a general perception is that recovering shark populations are to blame for recent increases in unprovoked shark incidents in Queensland and New South Wales^8^. By providing unique insight into past coastal ecosystem states, the QSCP data imply that increases in human–shark interactions are occurring at a time when shark populations are severely depleted compared to historical baselines. The timing of these observed declines precede previously reported collapses of coastal and pelagic apex sharks by several decades, and the magnitude of decline is either equal to or exceeding rates reported in coastal oceans elsewhere in the world^3,4,60^. Thus, shark populations within Australian coastlines may be predicated on a substantially shifted baseline. Promising signs of recovery have been reported from coastal shark populations that have undergone a history of severe exploitation in the Atlantic^61^, yet ongoing serial depletions of large sharks under the QSCP may impact upon local recovery of vulnerable and endangered coastal shark populations.

## Methods

### Historical reconstructions of fishing effort and shark catches

The Queensland Shark Control Program (QSCP) employs a series of baited drumlines and mesh nets adjacent to coastal beaches. The QSCP actively targets ‘dangerous’ and ‘potentially dangerous’ sharks^17^, specifically certain species of whalers (Carcharhinidae), tiger sharks (*Galeocerdo cuvier*), white sharks (*Carcharodon carcharias*) and hammerhead sharks (Sphyrnidae). As a historical record of shark catches, the QSCP is unique in that it represents a continuous documented long-term effort, and that both size and identity of sharks have been recorded since the onset of the programme. Data were accessed from the Queensland Shark Control Program (QSCP) from the State of Queensland, Australia through the Department of Agriculture and Fisheries (https://www.daf.qld.gov.au/fisheries/shark-control-program).

The QSCP initially used nets, although due to high levels of bycatch (predominantly turtles and dugongs^11^) and declines in the number of sharks caught in nets^17^, they were increasingly replaced with drumlines (Supplementary Figure 2). Nets are considered a passive way of capturing sharks moving across beaches, whereas baited drumlines actively target feeding sharks^11^. However, evidence suggests that nets may actively target sharks, as bycatch trapped in nets attract feeding sharks^17^. Previous studies indicate that different gear types select for different sharks: hammerhead sharks and rays were particularly vulnerable to net capture, whereas higher catch rates of tiger sharks were observed for drumlines^11^. Increases in sea surface temperatures 1962–2016 within regions is shown in Supplementary Figure 11.

Since the initiation of the QSCP in 1962, contractors have recorded the total length, sex, species and status (dead/alive) of each captured shark. Gear types have been standardised and largely unchanged since around 1992^11^. Nets (186 m in length, 6 m drop and 50 cm stretched mesh size^11^) are predominantly set parallel, and ~500–1000 m, from shore (water depth 7–12 m) depending on local bathymetric conditions (Supplementary Figure 1). Drumlines are positioned 500–1000 m from the shore and hooks (single 14/0 J hook^11^) are baited with either shark flesh (pre-2002) or mullet (post-2002). Nets and drumlines are checked by contractors 15–20 days each month^11^ (Supplementary Figure 1).

Species identification is generally considered unreliable prior to 1996, while data on species identification following a review of the QSCP in 1997 is considered more robust^9^. For long-term analysis (1962–2017) we selected four readily identifiable groups: (i) hammerheads (Sphyrinidae, readily identifiable by their flattened and laterally extended cephalofoil shaped head), (ii) requiem whaler sharks (Carcharhinidae), (iii) tiger sharks (*Galeocerdo cuvier*, readily identifiable by their large vertical body stripes and blunt head shape), and (iv) white sharks (*Carcharodon carcharias*, readily identifiable by their robust, large, conical snout and countershading, with a white underside and a grey dorsal area).

Effort data in the form of total number of nets and drumlines was reconstructed using historical records from contractor’s logbooks between 1962 and 2017 (Supplementary Figure 2). Historical effort records account for seasonal lifting of gear and swapping of gear between beaches during seasons to avoid bycatch of turtles and whales, and annual effort was adjusted to reflect these changes. Catch data was standardised by effort at each site to calculate catch per unit effort (CPUE^62^) for both gear types. Where catch records were unclear or uncertainty existed regarding number of drumlines or nets, beaches were excluded from the analysis. Similarly, with size data, individuals were excluded where contractors appeared to have recorded measurements in imperial units, or where sizes exceeded the maximum total length (TL_max_) for each group^46^. Prior to standardization of the programme in 1996, in some minor instances, gear type was recorded as ‘unknown’ where exact records were not kept (2.41% of total catch data). In these instances, catch from the ‘unknown’ category was assigned to either drumline or net gear types in proportion to the odds ratio of catch by drumlines or nets for each species by region combination.

### Statistical analysis

We modelled spatial and temporal variation in shark catches under the QSCP between 1962 and 2017. We used Bayesian generalised linear mixed models to model temporal change in catch as a function of time with nested random effects of region and sites within regions. As catch rates peaked during the warmer austral summer months (November to February), time was modelled following financial years (e.g. July 1962 to June 1963). Time was also treated as a random effect and its effect on catch was modelled with an order two random walk, which is equivalent to a cubic spline^63^. Gear (net, drumlines) was included in the model as a fixed effect, to account for differences in catchability between gear types. Catch was modelled with a negative binomial distribution, which was found to adequately account for over-dispersion in catch data. We included effort as an offset in the GLMM, thus the model’s predictions were for CPUE. Each group (hammerheads, whalers, tigers and white sharks) was modelled separately. Models were fit using the integrated nested Laplace approximations (INLA)^64^ in the R package ‘INLA’^65^.

Prior parameters for the random walk component were specified using the penalized complexity method which controls over-fitting of the temporal trend^66^. We used prior parameters of 0.1 and 0.01, though none of the models’ WAICs changed considerably with different choices. All other parameters were given vague (broad) priors. For each group two models were fitted, the first allowed the random walk to vary by regions (though the random walk component for all regions shared the same hyper-parameters), whereas the second had only an additive regional effect and a shared global random walk. We compared the two models using the WAIC^67^. We calculated annual zero-catch probability as the probability of catching no sharks at a given site per year. Initial catch rates were defined as the average CPUE for the first five years following the installation of gear at new beaches. To quantify the spatial scale of population declines, we explored changes in initial catch rates across all beaches between 1962 and 1998. We used Bayesian generalised linear mixed models with a random effect of region. Effort as an offset in the GLMM as above, and catch was modelled with a Poisson distribution.

### Long-term changes in size structure

Changes in the size of sharks over the long-term dataset (1962–2017) were explored for the four major groups (hammerheads, whalers, tigers and white sharks), and for short-term data for scalloped hammerheads and great hammerheads (1992–2017) using linear mixed effects models with gear and sex as fixed effects and site and region as nested random effects. We used previously published estimates of sizes at maturity of shark species from local studies^27,47^ to quantify changes in the probability of maturity in shark catches in the QSCP over the past two decades. While our estimates assume that size at maturity is fixed over time, we argue that this is a reasonable assumption in that sharks are less likely to exhibit rapid shifts in maturity due to their *K*-selected life-history strategies. Changes in the probability of catching mature individuals were assessed for either sex using binomial general linear models with site and region as nested random effects. Generalised linear models and GLMMs for maturity were fit using the package lme4^68^ and base package in R^69^.

### Regional movement patterns of coastal sharks

To assess potential large spatial scale effects of Queensland shark declines, we reviewed existing literature for shark movement data that included movements recorded within the Queensland coastline for shark species recorded in the QSCP catch. We then extracted a distance metric to represent the maximum movement recorded by an individual of each species. For both satellite and acoustic telemetry this comprised the shortest in-water distance between the furthest points of a minimum convex polygon for the widest-ranging tagged individual. For conventional mark-recapture (or re-sighting, in the case of photographic identification) studies using external tags, the greatest distance between initial capture and recapture point among individuals of each species was used. While the greatest distance moved by an individual of each species appears a relatively liberal representation of a species movement, we consider this metric to be somewhat conservative for a number of reasons. First, sample sizes in satellite tagging studies are generally small and deployments short. Therefore, only the longest tracks are more likely to accurately capture any seasonal movements undertaken by migratory species. Second, acoustic telemetry is limited by receiver placement and any movements beyond the range of receiver arrays are unknown. Third, mark-recapture studies are limited by spatial and temporal extent of recapture effort, and finally, we limited our search to movement data that was only within and/or overlapped the QSCP study areas.

## Electronic supplementary material

Supplementary Information

## Data Availability

Use of the Shark Control Program data is by courtesy of the State of Queensland, Australia through the Department of Agriculture and Fisheries (https://www.daf.qld.gov.au/fisheries/shark-control-program).
