# Supplementary material for: Decline of coastal apex shark populations over the past half century
Source: Commun Biol. 2018 Dec 13;1:223. doi: 10.1038/s42003-018-0233-1 (PMC6292889; doi:10.1038/s42003-018-0233-1)
Supplement: Supplementary file 1 — Supplementary Information [file 42003_2018_233_MOESM1_ESM.pdf]

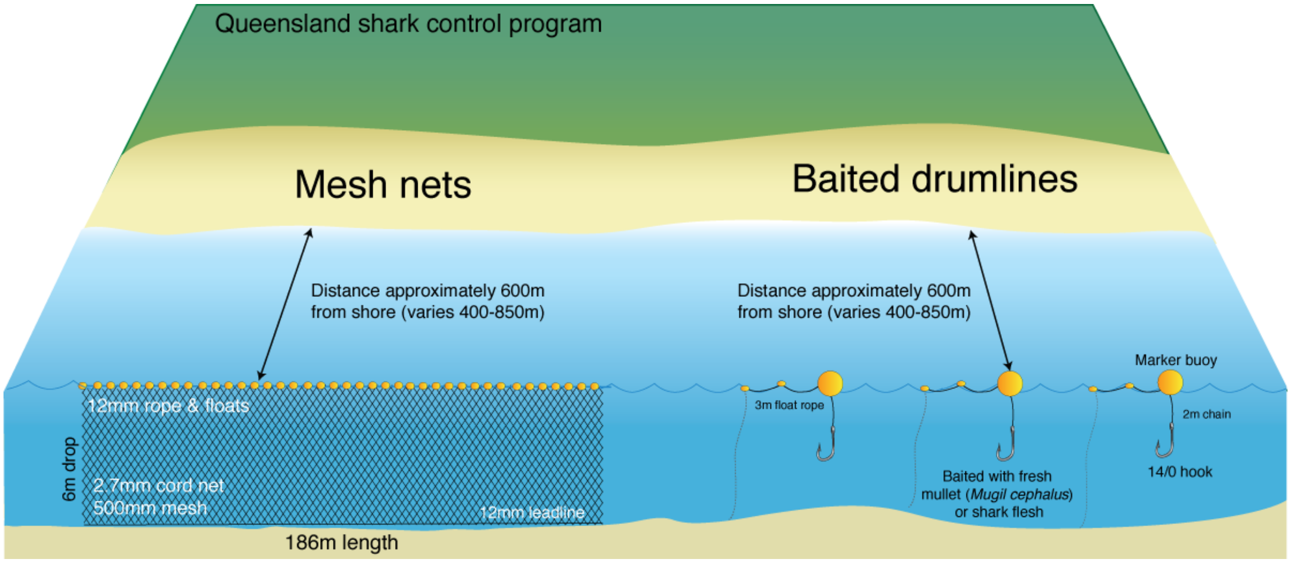


**Supplementary Figure 1** Arrangement of shark nets and baited drumlines used in the Queensland Shark Control Program from 1992 onwards

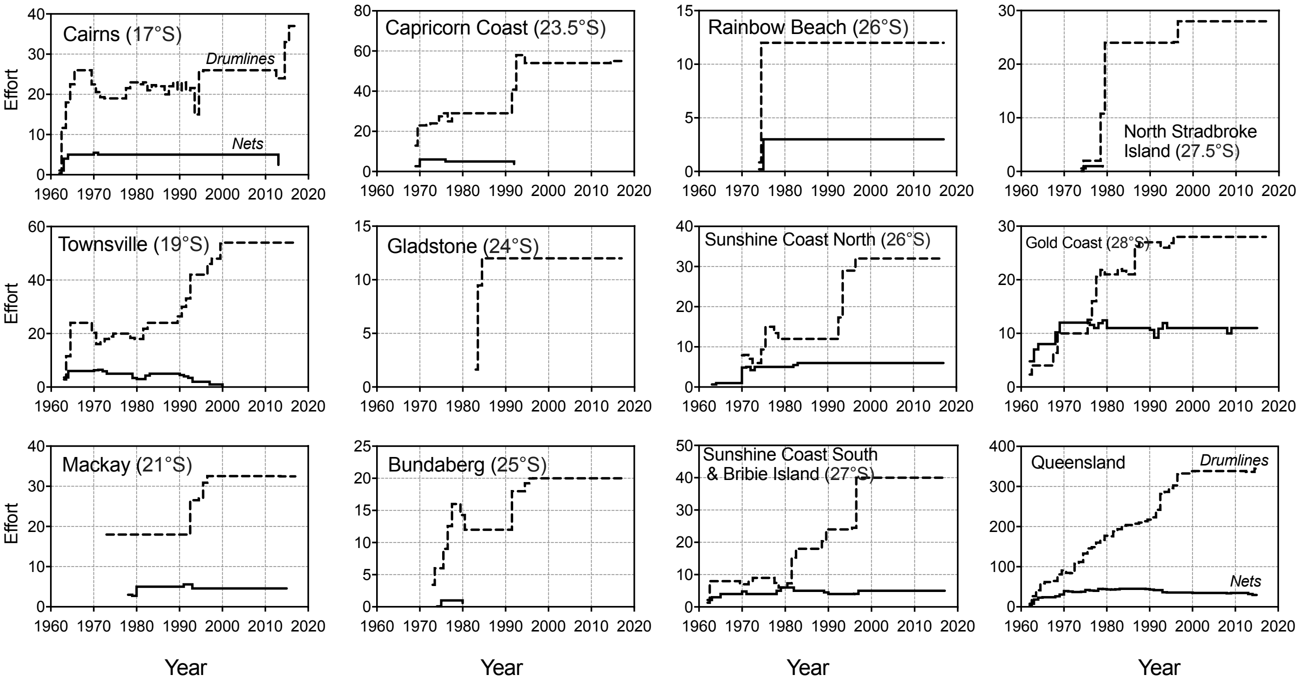


**Supplementary Figure 2** Temporal trends in effort per region for number of drum lines (dashed lines) and number of nets (solid lines) between 1962 and 2017.

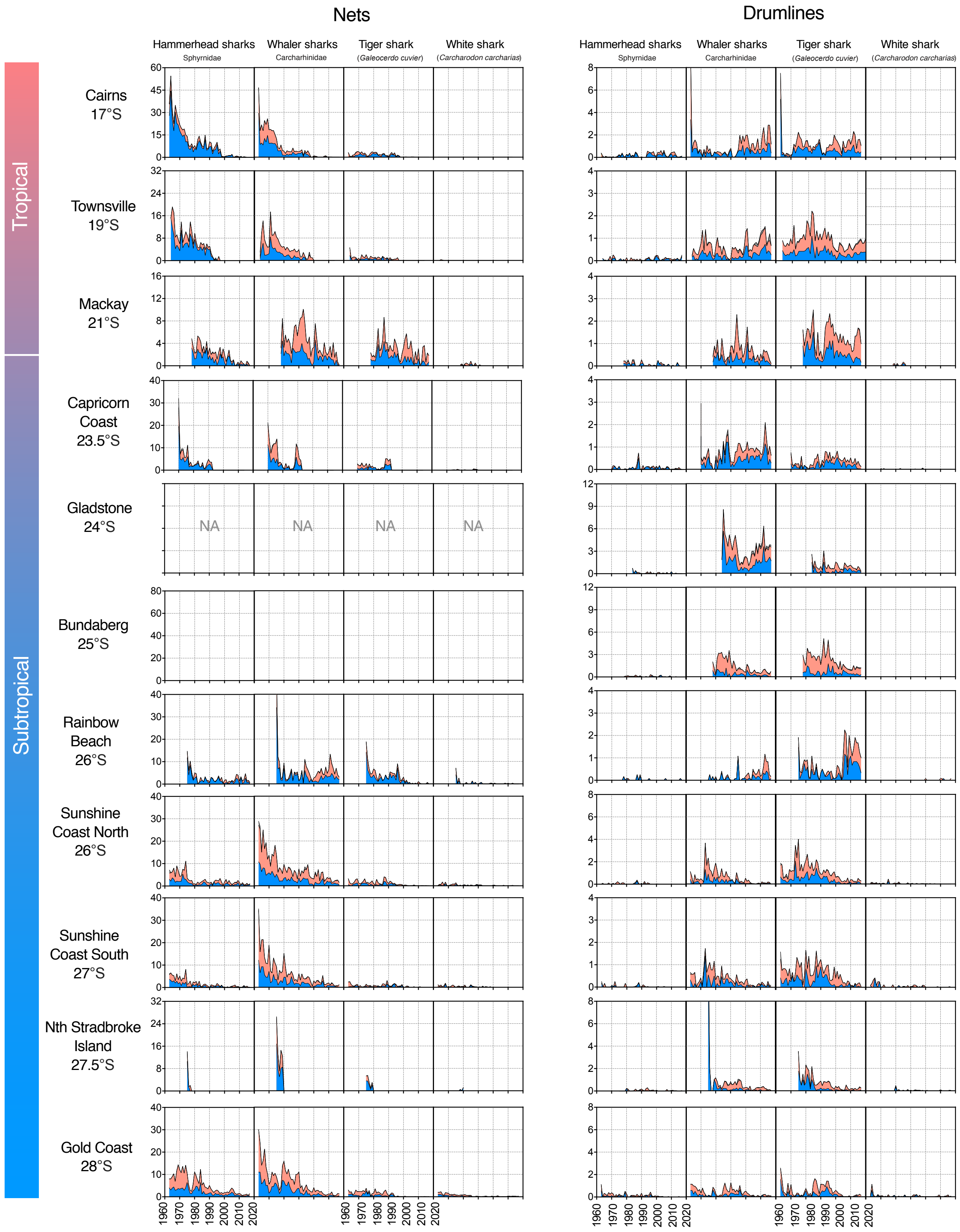


**Supplementary Figure 3** Stacked area plots of changes in catch per unit effort (CPUE) for each shark group (Sphyrinidae, Carcharhinidae, tiger sharks and white sharks) for each region separate by gear type (nets, drums) and gender (red = female, blue = male)

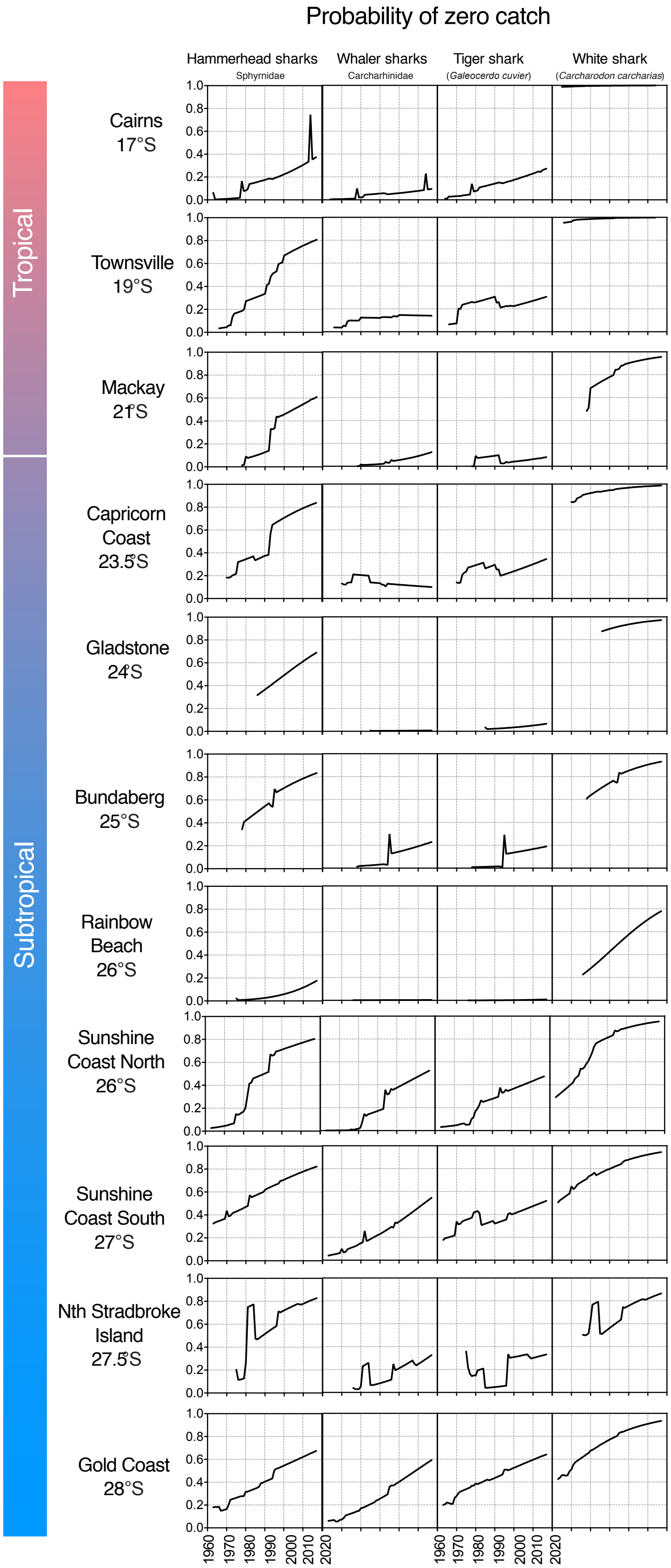


**Supplementary Figure 4** Average annual zero-catch probability for each shark group (Sphyrinidae, Carcharhinidae, tiger sharks and white sharks).

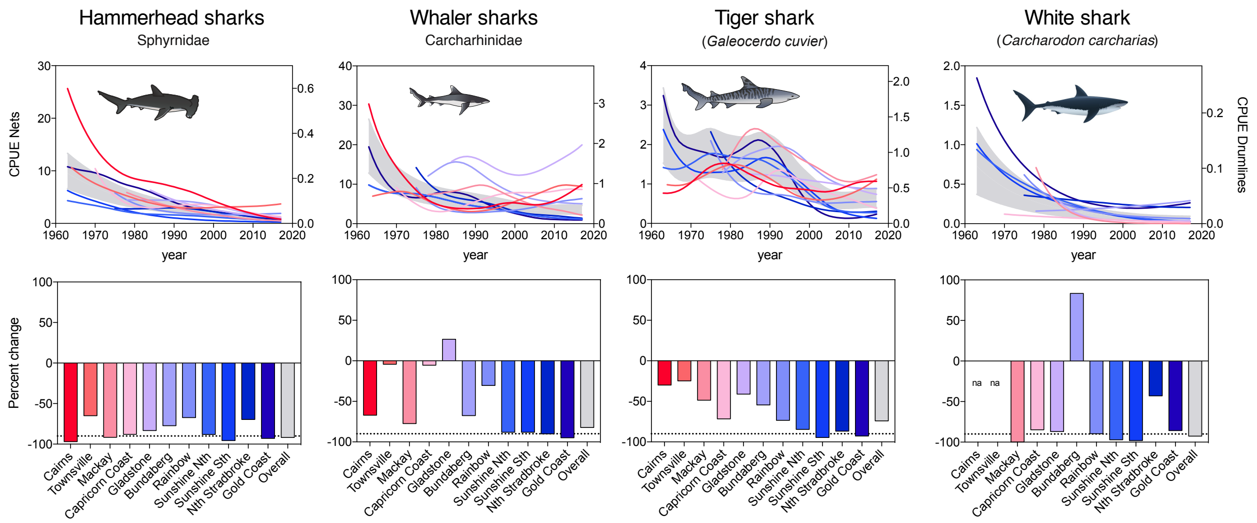


**Supplementary Figure 5** Regional trends in catch per unit effort (CPUE) in nets and drumlines with fits from Bayesian negative binomial generalised additive mixed effects models (upper panels) and percent change in CPUE (lower panels) for hammerhead sharks, whaler sharks, tiger sharks and white sharks. Percent change was defined as difference between the start of the time series to the end year (2017) within each region. White sharks were not recorded in catches at Cairns and Townsville (*na*).

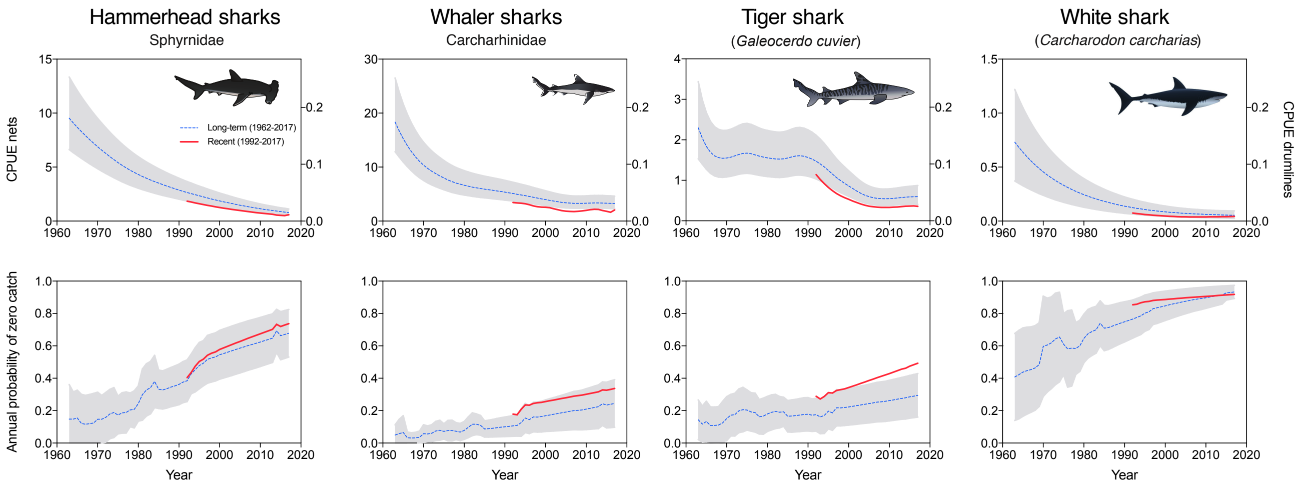


**Supplementary Figure 6** Comparison of Bayesian negative binomial generalised additive mixed effects models of catch per unit effort, and annual probability of zero catch using the long-term dataset (1962 - 2017) and the recent dataset following standardisation of the program (1992 – 2017, Table S3).

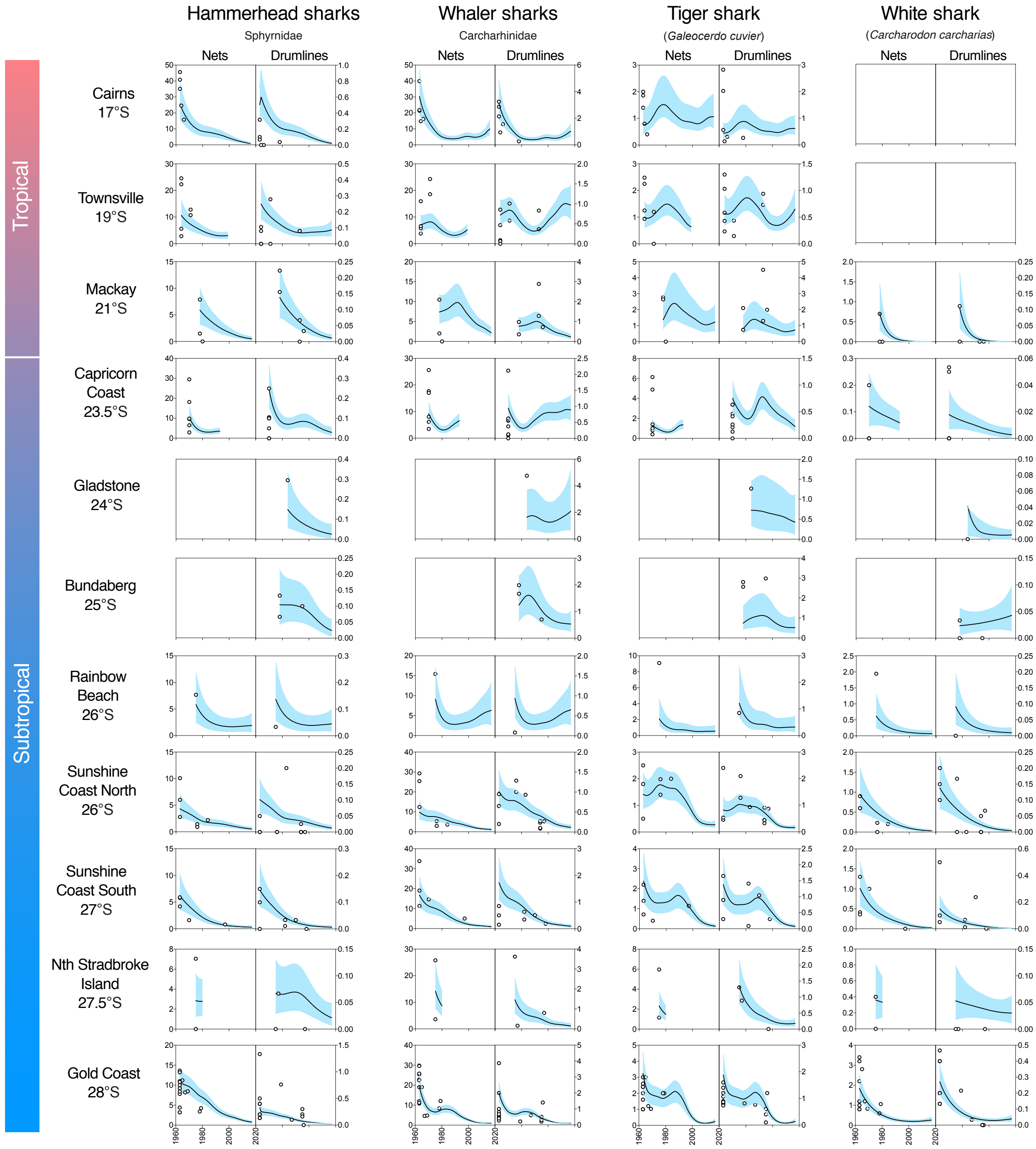


**Supplementary Figure 7** Temporal trends of catch per unit effort (CPUE) per region derived from Bayesian generalised additive mixed effects models (± 95% credibility intervals) for nets and drumlines. Circles are initial CPUE (defined as the average of the first five years of operation) for each beach within regions.

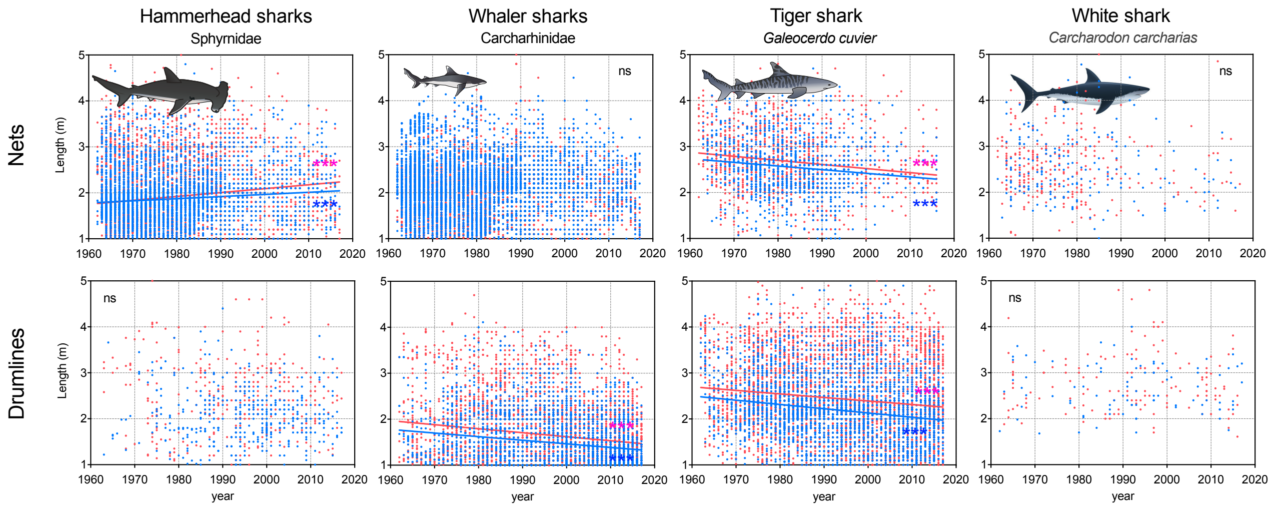


**Supplementary Figure 8** Changes in length of sharks between 1962-2017 for sharks caught in drum lines and nets separated by shark groups (Sphyrinidae, Carcharhinidae, tiger sharks and white sharks) and by gender.

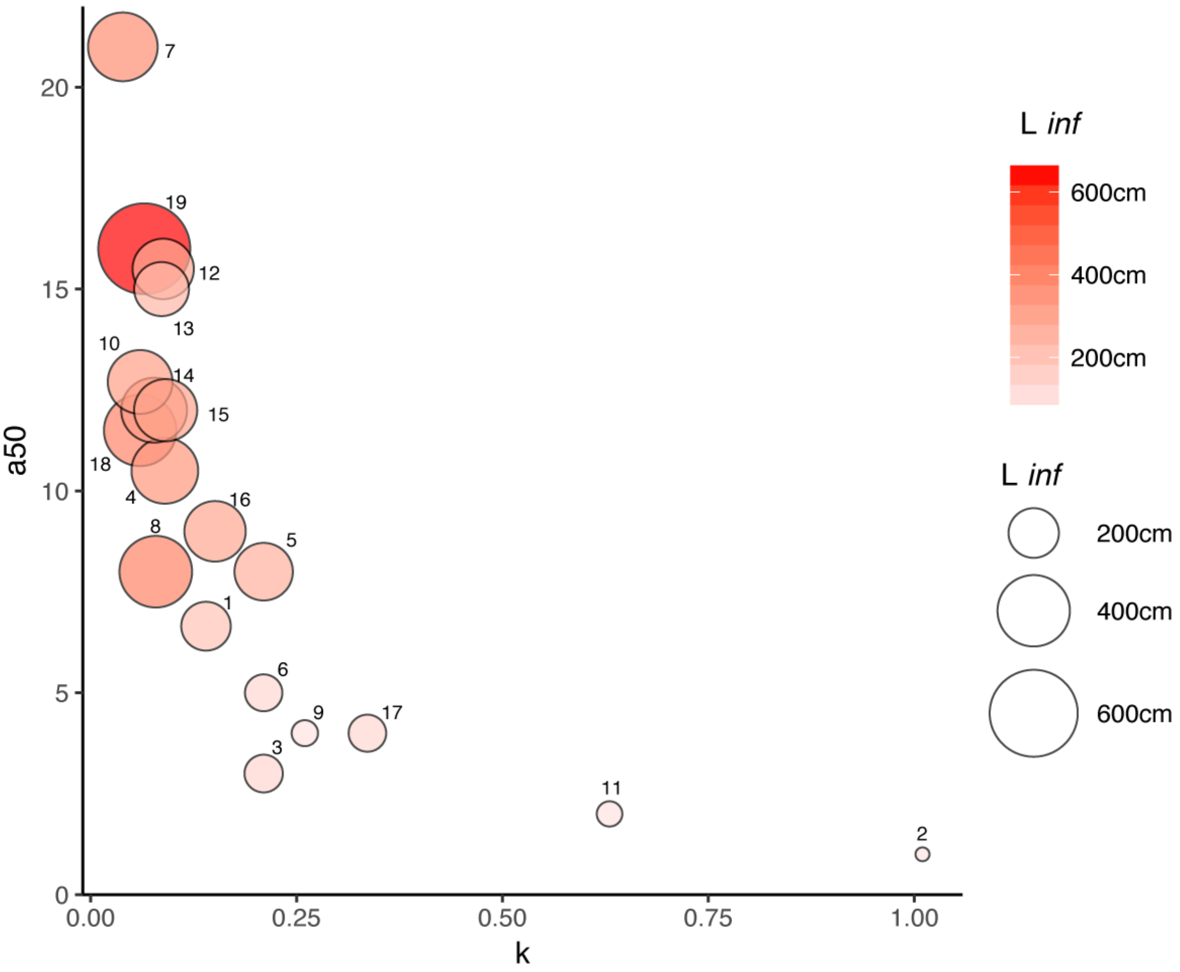


**Supplementary Figure 9** Age at maturation and maximum total size of common sharks caught in the QSCP, where k is the von Bertalanffy growth parameter for female sharks, a50 is age at 50% of maturity for female sharks, and L*inf* is the maximum total length (asymptotic length). 1) *Carcharhinus tilstoni*, 2) *Rhizoprionodon taylori*, 3) *Carcharhinus acronotus*, 4) *Carcharhinus leucas*, 5) *Carcharhinus limbatus*, 6) *Carcharhinus fitzroyensis*, 7) *Carcharhinus obscurus*, 8) *Sphyrna mokarran*, 9) *Carcharhinus macloti*, 10) *Negaprion acutidens*, 11) *Rhizoprionodon acutus*, 12) *Carcharhinus amboinensis*, 13) *Carcharhinus plumbeus*, 14) *Sphyrna lewini*, 15) *Carcharhinus falciformis*, 16) *Carcharhinus brevipinna*, 17) *Carcharhinus sorrah*, 18) *Galeocerdo cuvier*, 19) *Carcharodon carcharias*. Data from references [1-3].

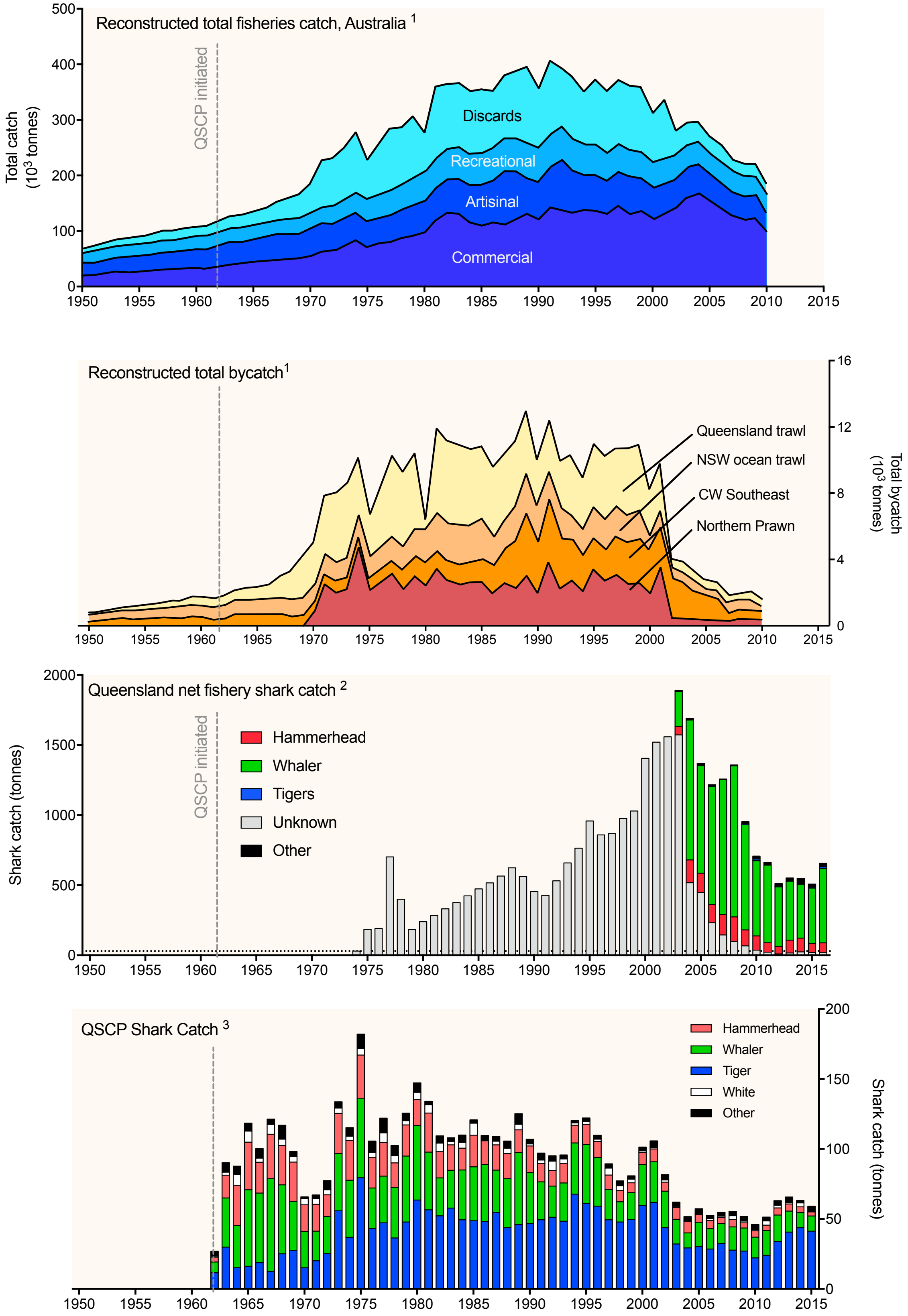


**Supplementary Figure 10** Historical regional fisheries in Australia 1950-2015. Dashed grey line is 1962 when the QSCP was initiated. Sources: [1] = [4], [2] = QFish (http://qfish.fisheries.qld.gov.au/), [3] = present study

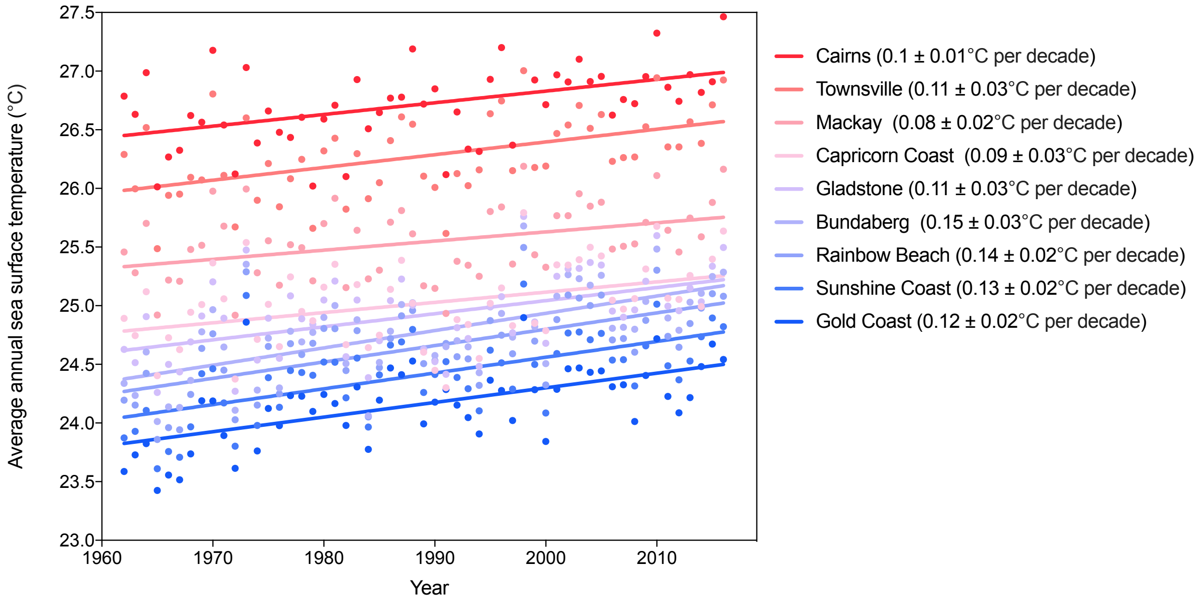


**Supplementary Figure 11** Linear increases in sea surface temperatures 1962 – 2016 within regions (data extracted from the Hadley Centre Sea Ice and Sea Surface Temperature data set [HadISST] for 1 degree grid cells and averaged across years). Warming trends across all regions were significant at $p < 0.005$.

**Supplementary Table 1** Species of sharks caught in the Queensland Shark Control Program (QSCP) between 1962 and 2017. Maximum total length and trophic level reported from [5], and % is proportion of total catch (1962-2017). * prior to 1992, hammerheads and whalers were not identified to species level. † Protected species under the Queensland Nature Conservation Act 1992

| **Group** | **Species** | **Max length (cm)** | **Trophic level** | **%** | **IUCN Red list status** |
|---|---|---|---|---|---|
| Hammerhead | Hammerhead shark * | na | na | 20.4 % | na |
| | *Eusphyra blochii* | 186 | 4.2 | < 0.1% | Endangered |
| | *Sphyrna lewini* | 430 | 4.1 | 1.5 % | Endangered |
| | *Sphyrna mokarran* | 610 | 4.3 | 0.6 % | Endangered |
| Tiger Shark | *Galeocerdo cuvier* | 750 | 4.5 | 26.3 % | Near Threatened |
| White Shark | *Carcharodon carcharias* † | 541 | 4.5 | 1.6 % | Vulnerable |
| Whalers | Whalers * | na | na | 23.4 % | na |
| | *Carcharhinus albimarginatus* | 300 | 4.2 | < 0.1% | Vulnerable |
| | *Carcharhinus altimus* | 300 | 4.5 | < 0.1% | Data Deficient |
| | *Carcharhinus* [illegible] | 255 | 4.1 | 0.1 % | Near Threatened |
| | *Carcharhinus amblyrhychos* | 255 | 4.1 | 0.6 % | Near Threatened |
| | *Carcharhinus amboinensis* | 280 | 4.3 | 0.6 % | Data Deficient |
| | *Carcharhinus brachyurus* | 325 | 4.5 | 0.2 % | Near Threatened |
| | *Carcharhinus brevipinna* | 300 | 4.2 | 2.3 % | Near Threatened |
| | *Carcharhinus cautus* | 150 | 4.3 | < 0.1 % | Data Deficient |
| | *Carcharhinus dussumieri* | 96 | 3.9 | 0.5 % | Near Threatened |
| | *Carcharhinus falciformis* | 350 | 4.5 | 0.1 % | Near Threatened |
| | *Carcharhinus fitzroyensis* | 135 | 4.1 | < 0.1% | Least Concern |
| | *Carcharhinus leucas* | 360 | 4.3 | 5.9 % | Near Threatened |
| | *Carcharhinus limbatus* | 275 | 4.4 | 0.2 % | Near Threatened |
| | *Carcharhinus macloti* | 110 | 4.2 | < 0.1 % | Near Threatened |
| | *Carcharhinus melanopterus* | 200 | 3.9 | 8.2 % | Near Threatened |
| | *Carcharhinus obscurus* | 420 | 4.3 | 0.6 % | Vulnerable |
| | *Carcharhinus plumbeus* | 180 | 4.5 | 0.5 % | Vulnerable |
| | *Carcharhinus sorrah* | 160 | 4.2 | 0.7 % | Near Threatened |
| | *Carcharhinus tilstoni* | 200 | 4.2 | < 8.2 % | Least Concern |
| | *Glyphis glyphis* † | 175 | 4.2 | < 0.1 % | Endangered |
| | *Loxodon macrorhinus* | 98 | 4.0 | < 0.1 % | Least Concern |
| | *Negaprion acutidens* | 380 | 4.1 | 0.4 % | Vulnerable |
| | *Prionace glauca* | 400 | 4.5 | 0.1% | Near Threatened |
| | *Rhizoprionodon acutus* | 175 | 4.3 | 0.1 % | Least Concern |
| | *Rhizoprionodon taylori* | 69 | 4.5 | < 0.1% | Least Concern |
| | *Triaenodon obesus* | 213 | 4.2 | 0.1 % | Near Threatened |
| Other | *Brachaelurus waddi* | 122 | 3.9 | < 0.1 % | Least Concern |

| | | | | | |
|---|---|---|---|---|---|
| | *Alopias vulpinus* | 549 | 4.5 | < 0.1 % | Vulnerable |
| | *Nebrius ferrugineus* | 320 | 4.1 | 1.9 % | Vulnerable |
| | *Chiloscyllium punctatum* | 132 | 4.1 | < 0.1% | Near Threatened |
| | *Hemipristis elongata* | 240 | 4.3 | < 0.1% | Vulnerable |
| | *Hemigaleus australiensis* | 110 | 4.4 | < 0.1 % | Least Concern |
| | *Eucrossorhinus dasypogon* | 366 | 4.0 | < 0.1 % | Least Concern |
| | *Isurus sp.* | 445 | 4.5 | 0.3 % | Vulnerable |
| | *Carcharias taurus* † | 330 | 4.5 | 0.5 % | Vulnerable |
| | *Stegastoma fasciatum* | 354 | 3.1 | 1.3 % | Unknown |
| | *Galeorhinus galeus* | 193 | 4.3 | 0.1 % | Vulnerable |
| | *Mustelus antarcticus* | 157 | 4.5 | < 0.1 % | Least Concern |

**Supplementary Table 2** Results from Bayesian mixed effects models of long-term changes in CPUE for major groups 1962 – 2017. Values are mean estimates with 95% CIs in brackets.

Sphyrinidae (hammerhead sharks) [1962-2017]

| Random effects | Variance (global model) | Variance (regional model) |
|---|---|---|
| Dispersion parameter | 0.6 (0.54 – 0.67) | 0.52 (0.46 – 0.59) |
| Temporal variance | 0.001 (0.001-0.008) | 0.22 (0.1 – 0.68) |
| Regional variance | 0.19 (0.08 – 0.58) | 0.05 (0.02 – 0.12) |
| Site variance | 0.17 (0.11 – 0.29) | 0.18 (0.12 – 0.29) |

| Fixed effects | Variance (global model) | Variance (regional model) |
|---|---|---|
| intercept | -2.61 (-2.28 – -2.94) | -2.69 (-2.34 – -3.05) |
| gear | 3.63 (3.53 – 3.74) | 1.05 (0.7 – 1.42) |

Carcharhinidae (whaler sharks) [1962-2017]

| Random effects | Variance (global model) | Variance (regional model) |
|---|---|---|
| Dispersion parameter | 0.72 (0.68 – 0.78) | 0.51 (0.47 – 0.55) |
| Temporal variance | 0.01 (0.001 – 0.07) | 0.06 (0.02 – 0.42) |
| Regional variance | 0.1 (0.03 – 0.55) | 0.15 (0.09 – 0.29) |
| Site variance | 0.24 (0.16 – 0.38) | 0.18 (0.12 – 0.28) |

| Fixed effects | Variance (global model) | Variance (regional model) |
|---|---|---|
| intercept | -0.34 (-0.63 - 0.01) | -0.51 (-0.20 – 0.76) |
| gear | 2.06 (1.97 – 2.15) | 1.76 (1.51 – 2.08) |

Tiger sharks (*Galeocerdo cuvier*) [1962-2017]

| Random effects | Variance – global model | Variance – regional model |
|---|---|---|
| Dispersion parameter | 0.56 (0.51 – 0.61) | 0.44 (0.4 – 0.49) |
| Temporal variance | 0.07 (0.03 – 0.21) | 0.001 (0.001 – 0.29) |
| Regional variance | 0.12 (0.03 – 0.6) | 0.18 (0.11 – 0.32) |
| Site variance | 0.39 (0.27 – 0.58) | 0.39 (0.27 – 0.59) |

| Fixed effects | Variance – global model | Variance – regional model |
|---|---|---|
| intercept | -0.36 (-0.01 – -0.68) | -0.6 (-0.81 – -0.28) |
| gear | 0.47 (0.37– 0.55) | -0.06 (-0.28 – 0.27) |

White sharks (*Carcharodon carcharias*) [1962-2017]

| Random effects | Variance – global model | Variance – regional model |
|---|---|---|
| Dispersion parameter | 0.85 (0.63 – 1.16) | 0.79 (0.58 – 1.09) |
| Temporal variance | 0.001 (0.001 – 0.02) | 0.36 (0.14 – 1.34) |
| Regional variance | 0.4 (0.15 – 1.6) | 0.01 (0.001 – 0.05) |
| Site variance | 0.09 (0.04 – 0.23) | 0.09 (0.04 – 0.22) |

| Fixed effects | Variance – global model | Variance – regional model |
|---|---|---|
| intercept | -3.79 (-3.3 – -4.39) | -3.85 (-3.33 – 4.41) |
| gear | 1.9 (1.7 – 2.1) | -1.93 (-1.41 – -2.5) |

**Supplementary Table 3** Results from Bayesian mixed effects models of long-term changes in CPUE for major groups 1992 – 2017. Values are mean estimates with 95% CIs in brackets.

Sphyrinidae (hammerhead sharks) [1992-2017]

| Random effects | Variance (global model) | Variance (regional model) |
|---|---|---|
| Dispersion parameter | 0.77 (0.7 – 0.86) | 0.74 (0.66 – 0.83) |
| Temporal variance | 0.01 (0.01-0.07) | 0.01 (0.01 – 0.07) |
| Regional variance | 0.45 (0.29 – 0.82) | 0.44 (0.28 – 0.81) |
| Site variance | 0.48 (0.37 – 0.65) | 0.48 (0.37 – 0.65) |

| Fixed effects | Variance (global model) | Variance (regional model) |
|---|---|---|
| intercept | -2.94 (-3.32 – -2.59) | -2.97 (-3.34 – -2.62) |
| gear | 2.91 (2.72 – 3.11) | 0.06 (-0.44 – 0.31) |

Carcharhinidae (whaler sharks) [1992-2017]

| Random effects | Variance (global model) | Variance (regional model) |
|---|---|---|
| Dispersion parameter | 0.71 (0.67 – 0.75) | 0.62 (0.58 – 0.67) |
| Temporal variance | 0.14 (0.07 – 0.31) | 0.06 (0.02 – 0.21) |
| Regional variance | 0.45 (0.27 – 0.91) | 0.5 (0.3 – 0.95) |
| Site variance | 0.62 (0.51 – 0.77) | 0.58 (0.48 – 0.73) |

| Fixed effects | Variance (global model) | Variance (regional model) |
|---|---|---|
| intercept | -0.62 (-0.99 – -0.21) | -0.67 (-1.06 – -0.24) |
| gear | 1.42 (1.27 – 1.57) | 0.88 (0.48 – 1.32) |

Tiger sharks (*Galeocerdo cuvier*) [1992-2017]

| Random effects | Variance – global model | Variance – regional model |
|---|---|---|
| Dispersion parameter | 0.62 (0.58 – 0.67) | 0.53 (0.49 – 0.58) |
| Temporal variance | 0.08 (0.05 – 0.17) | 0.16 (0.11 – 0.24) |
| Regional variance | 0.54 (0.34 – 1.05) | 0.64 (0.41 – 1.16) |
| Site variance | 0.68 (0.57 – 0.85) | 0.7 (0.58 – 0.87) |

| Fixed effects | Variance – global model | Variance – regional model |
|---|---|---|
| intercept | -0.69 (-1.12 – -0.23) | -0.77 (-1.26 – -0.26) |
| gear | -0.09 (-0.24 – 0.06) | -0.74 (-1.23 – -0.22) |

White sharks (*Carcharodon carcharias*) [1992-2017]

| Random effects | Variance – global model | Variance – regional model |
|---|---|---|
| Dispersion parameter | 0.66 (0.41 – 1.17) | 0.65 (0.4 – 1.2) |
| Temporal variance | 0.03 (0.01– 0.14) | 0.01 (0.01 – 0.09) |
| Regional variance | 1.06 (0.64 – 2.21) | 1.34 (0.8 – 2.87) |
| Site variance | 0.35 (0.2 – 0.71) | 0.32 (0.17 – 0.68) |

| Fixed effects | Variance – global model | Variance – regional model |
|---|---|---|
| intercept | -4.52 (-5.5 – -3.66) | -4.85 (-6.12 – -3.8) |
| gear | 1.31 (0.92 – 1.7) | -3.54 (-4.85 – -2.45) |

**Supplementary Table 4** Results of linear mixed effects models for changes in size through time among shark groups. Model simplification from full model (year * gear * sex) was based on Akaike Information Criterion (AIC).

Sphyrinidae [1962-2017]

| | **SS** | **MSS** | **DF** | **DenDF** | **F** | **P** |
|---|---|---|---|---|---|---|
| year | 24.153 | 24.153 | 1 | 8220.4 | 53.281 | <0.001 |
| gear | 15.95 | 15.95 | 1 | 4823.2 | 35.186 | <0.0001 |
| sex | 36.121 | 36.121 | 1 | 9691.6 | 79.683 | <0.0001 |
| gear:sex | 10.629 | 10.629 | 1 | 9630.2 | 23.448 | <0.0001 |

Carcharhinidae [1962-2017]

| | **SS** | **MSS** | **DF** | **DenDF** | **F** | **P** |
|---|---|---|---|---|---|---|
| year | 15.2172 | 15.2172 | 1 | 17343 | 45.868 | <0.0001 |
| sex | 1.9214 | 1.9214 | 1 | 20008 | 5.792 | <0.0001 |
| gear | 4.9642 | 4.9642 | 1 | 17770 | 14.963 | <0.0001 |
| year:gear | 5.1763 | 5.1763 | 1 | 17706 | 15.602 | <0.0001 |
| year:sex | 1.7281 | 1.7281 | 1 | 20008 | 5.209 | <0.05 |

Tiger sharks (*Galeocerdo cuvier*) [1962-2017]

| | **SS** | **MSS** | **DF** | **DenDF** | **F** | **P** |
|---|---|---|---|---|---|---|
| year | 147.688 | 147.688 | 1 | 10342.6 | 245.949 | <0.0001 |
| gear | 59.739 | 59.739 | 1 | 7193.8 | 99.484 | <0.0001 |
| sex | 65.014 | 65.014 | 1 | 12508.2 | 108.27 | <0.0001 |
| gear:sex | 7.713 | 7.713 | 1 | 12517.7 | 12.844 | <0.0001 |

White sharks (*Carcharodon carcharias*) [1962-2017]

| | **SS** | **MSS** | **DF** | **DenDF** | **F** | **P** |
|---|---|---|---|---|---|---|
| gear | 45.028 | 45.028 | 1 | 9108.2 | 133.67 | <0.0001 |

Scalloped hammerhead (*Sphyrna lewini*) [1995-2017]

| | **SS** | **MSS** | **DF** | **DenDF** | **F** | **P** |
|---|---|---|---|---|---|---|
| year | 3.2317 | 3.2317 | 1 | 662.06 | 11.58 | <0.0001 |
| sex | 0.5766 | 0.5766 | 1 | 671.06 | 2.066 | >0.1 |
| gear | 1.6952 | 1.6952 | 1 | 374.34 | 6.074 | <0.05 |
| sex:gear | 1.8956 | 1.8956 | 1 | 668.42 | 6.792 | <0.001 |

Great hammerhead (*Sphyrna mokarran*) [1995-2017]

| | **SS** | **MSS** | **DF** | **DenDF** | **F** | **P** |
|---|---|---|---|---|---|---|
| year | 4.2379 | 4.2379 | 1 | 292.79 | 14.8909 | <0.0001 |
| sex | 1.9552 | 1.9552 | 1 | 292.21 | 6.8701 | <0.001 |
| gear | 1.9552 | 1.9552 | 1 | 292.21 | 6.8701 | <0.001 |
| year:sex | 4.641 | 4.641 | 1 | 290.48 | 16.3073 | <0.0001 |
| year:gear | 0.2414 | 0.2414 | 1 | 289.81 | 0.8482 | >0.1 |
| sex:gear | 1.9552 | 1.9552 | 1 | 292.21 | 6.8701 | <0.001 |
| year:sex:gear | 1.9537 | 1.9537 | 1 | 292.2 | 6.8648 | <0.001 |

**Supplementary Table 5** Results of binomial probability general linear models for changes in maturity through time among shark groups

Binomial GLM Scalloped hammerhead (*Sphyrna lewini*) [1995-2017]

| | **Estimate** | **SE** | **z value** | **P** |
|---|---|---|---|---|
| year | -0.07602 | 0.01618 | -4.698 | <0.001 |

Binomial GLM Great hammerhead (*Sphyrna mokkaran*) [1995-2017]

| | **Estimate** | **SE** | **z value** | **P** |
|---|---|---|---|---|
| year | -0.04563 | 0.02257 | -2.021 | <0.05 |

Binomial GLM Tiger shark (*Galeocerdo cuvier*) [1995-2017]

| | Estimate | SE | z value | P |
|---|---|---|---|---|
| year | -0.031625 | 0.005666 | - 5.581 | <0.0001 |

**Supplementary Table 6** Alternative hypothesis to account for the long-term decline in shark populations in the QSCP data

| |
|---|
| **Hypothesis 1: Environmental changes (increasing SST) are responsible for long-term declines in shark populations** |
| Theoretically, shifts in environmental conditions (rainfall and temperature) could affect shark abundance and distribution over decadal scales. On the Queensland coastline, rainfall and river flow variability are strongly influenced by El Nino Southern Oscillation events and the Pacific Decadal Oscillation, resulting in periodic wet seasons and dry seasons. Analysis of 20$^{th}$ century patterns of rainfall indicate that there appears to be no overall trend toward wetter or drier conditions[19], precluding long-term changes in rainfall as a driver of declining shark populations. Analysis of long-term trends of sea surface temperature (SST) over the past five decades (Figure S11) indicates that all QSCP regions have experienced a significant warming trend of between 0.08 to 0.14°C per decade. While long-term increases of temperatures in the past decades occur at a time when shark population are in decline, SST is unlikely to be a primary driver of shark declines, as declines in shark populations were non-linear, and declined rapidly in the 1960's to 1970's (Figure 2) under relatively slow linear increases in temperature (Figure S11). Concurrent warming patterns in SST could theoretically result in changes in latitudinal distribution shifts (e.g. [20]), yet it seems unlikely that relatively minor changes in SST (~0.1 °C per decade) would account for such substantial non-linear declines in shark populations. Warming SST in high latitudes regions could theoretically drive distribution shifts, resulting in more incursions of tiger sharks that prefer tropical and warm-temperate oceans[5]. Yet, the strongest declines in tiger shark CPUE were recorded at high latitude regions (Fig S5). While environmental fluctuations can influence the distribution and habitat use of coastal sharks[21], it seems unlikely that long-term shifts in the environment are responsible for rapid declines in coastal shark populations. |
| **Hypothesis 2: Long-term changes in prey abundance have resulted in declines in shark populations** |
| Seasonal abundances and habitat use of apex sharks can be linked to both water temperature and availability of prey[22,23]. However, there is no clear evidence of declines in prey coinciding with the rapid declines in CPUE in the 1960's and 1970's (Figure 2). Large apex sharks exhibit a high degree of omnivory: tiger sharks feed on sharks, rays, bony fishes, marine mammals, tortoises, seabirds, sea snakes, squids, gastropods, crustaceans, including carrion, garbage, cans, and pieces of metal[5], white sharks feed on a bony fishes, sharks, rays, seals, dolphins and porpoises, sea birds, carrion, squid, octopi and crabs[5], great hammerhead sharks feed on stingrays and other batoids, groupers and sea catfishes, but also small bony fishes, crabs, squid, other sharks, rays, and lobsters [5]. Further, large apex sharks often exhibit facultative scavenging behaviour, often feeding on weak and diseased individuals rather than actively hunting prey[23,24]. Given such a high degree of omnivory and facultative scavenging, it seems unlikely that declines in prey items or shifts in food-web structure would account for such substantial and rapid declines in shark populations (Figure 2). Furthermore, declines in shark populations in the 1960's and 1970's were spatially consistent across the Queensland coastline (Figure |

S5), and occurred during a time when coastal human population growth was comparatively minor compared to current human population size.

**Hypothesis 3. Sharks are learning to avoid nets and drums**

An alternative explanation may be that declines in CPUE within beaches result from sharks "*…learning that nets and drumlines placed in their local areas represent an obstacle and actively avoid(ing) them* [25]". While it has been broadly speculated that sharks are capable of some degree of learning (see [26] for a concise review), it seems improbable that avoidance behaviour can account for the rapid declines observed across multiple taxa across the length of the Queensland coastline (Fig S5). Such learned avoidance of QSCP program locations is particularly unlikely in sharks that exhibit widespread movement patterns at regional scales (e.g. *Galeocerdo cuvier* and *Carcharodon carcharias*, Fig 5). The QSCP uses two methods of shark control: nets that passively trap a broad range of shark species, and baited drumlines that actively target large sharks[27]. While some degree of operant conditioning could occur from baited hooks[26], high rates of bait scavenging in shark control programs (~60% of hooks within a single day of deployment[28]) implies that sharks are attracted to, and are actively targeting baited drumlines. Contractors notes from the early years of the QSCP (Sunshine Coast, 1963) suggest that drumline installations actively attract sharks to beaches where gear is installed rather than displace them:

> "*… thirty five feet of shark were taken on one drumline when hooks were shackled back to back at the end of the trace. An 11 foot whaler had swallowed both hooks and was attacked by a 13 foot tiger which also became hooked. Both were attacked by other sharks and another 11 foot whaler took one of the free hooks*" [29]

There are several lines of evidence that argue against net avoidance: i) declines in catches in shark netting programs occur even when nets have been set randomly within a beach and on different days[30], ii) nets may be set in turbid coast water, where sharks are unable to visually avoid entanglement, and iii) learned behaviour is unlikely to be intergenerational, as sharks would die without transferring the knowledge that nets are dangerous (Wallett 1983, cited in [31]). The evidence from the QSCP argues against the hypothesis of learned avoidance, in that sharks and bycatch trapped in nets actively attracts larger sharks decades after the gear was first installed:

> "*On 3 June 1975 two tiger sharks and three whaler sharks were caught at Bundaberg and all were found in close proximity to a meshed and severely mauled dugong*" [32]

> "*On 7 November 1977 a 5.1 m white pointer shark was caught at the Gold Coast while attacking a meshed dolphin. The stomach of the shark contained the caudal third of the dolphin*" [32]

> "*On 11 June 1985 seven tiger sharks and one white pointer shark were caught at Mackay and all were found in close proximity to a meshed dolphin*" [32]

**Hypothesis 4. Sharks are actively avoiding coastal regions with high human activity**

As shark control programs are set adjacent to major metropolitan areas that have grown rapidly and become increasingly degraded and noisy in recent years, large apex sharks may be actively avoiding such busy coastal regions. Indeed, such displacement in response to increased boat traffic has been observed in a broad range of cetaceans[33,34] and large predatory fish[35]. However, acoustic tagging studies of mesopredator sharks (*Triaenodon obesus* and *Carcharhinus amblyrhynchos*) showed no strong evidence of a link between spatial habitat use and intensity of human activities[35]. Conversely, large apex sharks with generalist diets may be attracted to coastal habitats while scavenging for food sources, and port areas are recognised as having a higher risk of shark attacks[36]. Such evidence is supported by studies that indicate that large apex sharks (specifically *G. cuvier*) may be attracted to coastal areas by increased levels of maritime traffic [37,38]. The use of baited drumlines are designed to attract sharks to control programs, and studies have documented the change movement patterns and behaviour of sharks in response to baiting and feeding [39-41].

While increased urbanisation of the Queensland coastline is likely to have affected coastal ecosystems and food-webs, the rapid declines in shark catches in the early decades of the QSCP (1960's and 1970's) occurred at a time when coastal development was low, prior to recent coastal migration and substantial tourism development in the 1990's and onwards [42-44]. Further, a review of current and historical distributions of marine mammal populations in Moreton Bay suggests that Queensland coastal ecosystems support a remarkably diverse and abundant marine mammal fauna, despite substantial population increases and coastal development over the past 50 years [45].

**Supplementary Table 7** Summary of commercial and recreational fisheries for sharks operating in Queensland and adjacent jurisdictions. Lack of detailed data on species identification and shark numbers within historical fisheries records makes it difficult to assess the impacts of commercial and recreational fisheries on shark populations. We collated local and adjacent fisheries to determine (where possible) species composition of shark catches and historical estimates of fisheries intensification over the past 50 years to contrast with data collected from the QSCP.

**Commercial fisheries**

<u>Queensland shark fisheries</u>

The majority of commercial shark product caught in Queensland, accounting for ~94% of all sharks, comes from the East Coast Inshore Fin Fish Fishery (ECIFFF)[46]. Shark fisheries had been operating for some years on some parts of the East Coast, but numbers of the shark catches were only recorded from 1974–75 onwards. Catches of sharks may have been very small prior to the mid-1970s, when many gillnet fishers using heavy nets were practically able to fish only rivers and estuaries[1]. Major shifts in net technology occurred in the 1970s, with the take-up of lightweight untarred nylon nets which allowed gillnet fishers to fish further from shore on the Queensland East Coast and actively target sharks[1] (Figure S11). The increase of shark catch from 1994 – 2003 from 319 to 1252 tonnes (Figure S10) raised concern from fishery and marine park managers[46,47]. Since 2003 the catch of sharks has fallen (Figure S10) primarily due to shark-specific measures implemented by fishery managers including restriction of license retention, possession limits and fishery-wide catch limits, along with a maximum size limit of 150 cm to protect large breeding females. While historical data on species composition is unavailable prior to 2003 (Figure S10), fisheries observer data indicates that blacktips (*Carcharhinus tilstoni*) and scalloped hammerheads (*S. lewini*) dominated commercial catches (32% and 18% respectively), while great hammerheads (*S. mokarran*) and neonate tiger sharks (*G. cuvier*) were less frequent (2.9% and 0.2% respectively)[46]. Based on the average catch of sharks from 1990 to 2005 (793 tonnes per year), the estimated catch of scalloped hammerheads (*S. lewini*) was 143 tonnes per year[47]. Later fishery observer surveys between 2006 and 2009 indicate that while whaler sharks (Carcharhinidae) formed 94.5% of catch by abundance, scalloped (*S. lewini*) and great (*S. mokarran*) hammerheads were the third and fourth most targeted species by biomass (9.7 and 6.8% respectively)[48]. Estimates of historical shark finning are largely unknown. Around 5.3 to 5.8 tonnes per year in the late 1990's was derived from shark landings in Queensland, although this is likely to be an underestimate[49].

The East Coast Otter Trawl Fishery (ECOTF) targets a range of prawns, scallops, bugs and squid species across on the eastern Queensland coastline[50]. In terms of the volume of product caught, the fishery is the largest in Queensland, operating across estuaries, coastal and oceanic waters, and encompassing approximately 57% of the Great Barrier Reef Marine Park[51]. Recent bycatch records indicate interactions with scalloped hammerheads (*S. lewini*) [50]. Prior to the introduction of bycatch reduction devices and mitigation measures in 2005, given the size of the fishery, substantial unreported numbers of sharks may have been caught as bycatch. As with nearly all fisheries, historical catch data and effort data from the onset of the QSCP are unavailable, although reconstructed fisheries estimates indicates increasing rates of

| |
|---|
| bycatch in the 1970's [4] prior to a reduction in the early 2000's with the onset of bycatch reduction (Figure S10). |
| East coast Australian fisheries<br>The Eastern tuna and billfish (ETBF) fishery operates using longline and minor line (including hand line, troll, rod and reel) fishing gear. It is unlikely that the fishery has strong impact on coastal sharks, as the shark catch in the fishery has historically been dominated by blue shark (*Prionace glauca*), oceanic white tip (*Carcharhinus longimanus*), and shortfin mako (*Isurus oxyrinchus*) that are uncommon in the QSCP[52], although tiger sharks (*G. cuvier*) and hammerheads (*S. lewini* and *S. mokkaran*) were historically finned along with a range of whaler sharks (Carcharhinidae)[49]. Logbook data from 1995 - 1996 indicates that sharks represent a significant portion (6.4 - 13.6%) of the catch in the ETBF, with 33 – 53% of sharks retained or finned, although this may be an underestimate due to under-reporting of catch[49]. Australian and Japanese longline fisheries targeting tunas and billfish in the Australian Exclusive Economic Zone (EEZ) have been ongoing since the 1960's. These fisheries are unlikely to impact coastal shark populations as they target pelagic sharks (primarily *Prionace glauca,* ~85% of total catch) that are uncommon in coastal Queensland waters and the QSCP catch[53]. Further, other line fisheries that operate in eastern Australian coastal regions are highly selective, and catch of non-target shark bycatch is relatively infrequent[54].<br><br>The Ocean Trap and Line fishery (OTLF) is a multi-method, multi species fishery targeting demersal and pelagic fish along the entire New South Wales coast in coastal and open ocean waters. The fishery targets sandbar sharks (*Carcharhinus plumbeus*, ~33% of total catch), dusky whaler (15.2%) and spinner sharks (10.6%) that occur within the QSCP long-term catch as 'whalers' (Carcharhinidae)[55,56]. While tiger and hammerhead sharks are a comparatively minor component of the OTLF shark catch (5.9% and 3.7% respectively), specific targeting of large individuals for shark finning prior to a prohibition in 2011 may have historically impacted upon shark populations. Long-term records since 1974 show that total catch rates have remained stable over the 30-year period, with reported shark catches from 1990 onwards showing a decline in shark catch[57]. |
| Regional SE Pacific shark fisheries<br>At a regional scale, shark fisheries in the Pacific were generally low until the late 1970's, and intensification of targeted fisheries for shark finning increased in the 1980's[56,58]. Prior to the introduction of pelagic long-line and industrial fisheries in the mid 1980's – mid 1990's, shark fisheries in the SW Pacific region were mostly small-scale artisanal fisheries relying on traditional fisheries methods[58-61]. While it is plausible that some declines occurred during oceanic excursions within the region (Figure 5), it seems unlikely that regional Pacific shark fisheries have a substantial impact on the observed declines, particularly in the early years of the program. |
| **Recreational fisheries**<br>The extent to which recreational fisheries have impacted upon coastal shark populations is unclear. Commercial production is reported by weight, while recreational fisheries is typically reported in terms of numbers, rendering comparisons among sectors difficult[62]. While Queensland's human population in the 1960's and 1970's was relatively stable, increases large shark fishing for white sharks occurred in the 1950's following popularisation of "shark hunting" and big game fishing[63]. Recreational spearfishing for |

grey nurse sharks (*Carcharias taurus*) on the eastern Australian coastline in the 1950's and 1960's resulted in near extirpation of local populations.

Current recreational fisheries largely target smaller species with an average weight of just 15kg[64], although some sport fishing does target large oceanic and pelagic sharks that overlap with QSCP catches. Leigh[1] argues that the impact of recreational fisheries on Queensland's shark populations is comparatively minor, as most (88.6%) of sharks caught are released. Considering that common fishing gear is designed to catch sharks <1m (~0.25kg to 6kg in weight), the average retained recreational catch of sharks is estimated to be ~12-75 tonnes[1]. Fisheries independent estimates of recreational fishing indicate that whalers (Carcharhinidae) form 86.8% of the catch in the Great Barrier Marine Park, with hammerheads (*S. lewini* and *S. mokkaran*) forming 7.8% of catch, which is broadly similar to the composition of shark catches in the ECIFFF[62].

Shark catches from sport fishermen off New South Wales between 1959-1979 indicates that most of the catch (>50%[65]) was composed of the pelagic shortfin mako (*Isurus oxyrinchus*) and blue sharks (*Prionace glauca*). Coastal sharks including tiger, and hammerhead sharks were historically targeted in lower numbers (<20% of total catch[65]). White sharks were rare in the 1979-1982 landings (0.4%) but accounted for 7% of all landings in 1959-1979, suggesting early population decline[65]. Shark catches from sport fishermen from Queensland in the late 1990's indicates that recreational catch is also primarily composed of the pelagic shortfin mako (*Isurus oxyrinchus*, 41%), with tiger (17.4%), whaler (16.7%), hammerhead (13%) blue sharks (12%), and that 80-100% of sharks were released, with the exception of tiger sharks, where larger individuals were kept for weighing[66]. On the Great Barrier Reef, line fisherman mostly target smaller reef sharks (*Carcharhinus melanopterus* and *Carcharhinus amblyrhynchos*, 56% catch), with large apex sharks such as tigers (1.5%) and hammerhead (7.8%) comparatively rare[62].

**NSW Shark meshing program**

The New South Wales (NSW) government has operated a shark netting program since 1937 in Sydney (33.8°S), and since 1949 in Newcastle (32.9°S) and Wollongong (34.4°S). Continuous data from the early years of the program is not available, and analysis is limited to decadal trends[67]. Records of catches between 1950 – 2010 indicated three main shark taxa: hammerhead sharks (*Sphyrna* spp.), whaler sharks (*Carcharhinus* spp.) and Australian angel sharks (*Squatina australis*)[67]. Catch rates of sharks in the NSW program are substantially lower than that of the QSCP. Similar to the QSCP, catches of large white sharks (Carcharodon carcharias) and tiger sharks (Galeocerdo cuvier) declined over time, although the magnitude of the decline was substantially less than the QSCP[67]. Tiger sharks were stable across the first 60 years of data, however a significant decline was observed in the last 20 years, and cyclical peaks in catch that were observed in catch rates ceased after 40 years of catch. Catch rates of white sharks (*C. carcharias)* in shark meshing programs is low (~six white sharks per year) but significantly reduced from the 1950's[67]. While the shark meshing program is located a substantial distance from the southernmost beaches of the QSCP (~700km), migration of tiger[10] and white sharks[8] between tropical and subtropical waters (Figure 5) implies that shark catches in the NSW shark meshing programs may be contributing to the declines observed in the Queensland Shark Control Program".

**Supplementary Table 8**. Summary of movement studies for 13 shark species that included movements of individuals within South East Queensland and have also been recorded in the QSCP. *S = satellite tags; A = acoustic tags; R = archival (non-transmitting) tags; T =conventional external tags (mark-recapture); P = conventional external tags (photo-ID re-sighting). §Total number of individuals tagged. Figures in parentheses refer to number of tagged individuals that were recaptured or re-sighted with conventional external tags.

| **Species** | **Method*** | **Number §** | **$D_{max}$** | **Source** |
|---|---|---|---|---|
| *Carcharodon carcharias* | S | 25 | 3665 | [6] |
| *Carcharodon carcharias* | S, R, T | 405 (1) | 3298 | [7] |
| *Carcharodon carcharias* | S, A | 10 | 2484 | [8] |
| *Carcharhinus leucas* | A | 114 | 1985 | [9] |
| *Galeocerdo cuvier* | S | 18 | 1832 | [10] |
| *Galeocerdo cuvier* | S, A, P | 34 | 1442 | [11] |
| *Galeocerdo cuvier* | S | 10 | 1259 | [12] |
| *Carcharias taurus* | S, A | 15 | 1550 | [13] |
| *Carcharias taurus* | P | 931 (386) | 1260 | [14] |
| *Carcharias taurus* | A | 21 | 1050 | [15] |
| *Carcharias taurus* | T, P | 24 (14) | 681 | [16] |
| *Carcharhinus sorrah* | T | 436 (7) | 683 | [17] |
| *Carcharhinus tilstoni* | T | 957 (35) | 309 | [18] |
| *Carcharhinus brevipinna* | T | 643 (46) | 169 | [17] |
| *Carcharhinus amblyrhynchos* | A | 9 | 134 | [17] |
| *Carcharhinus amboinensis* | T | 410 (33) | 123 | [17] |
| *Rhizoprionodon taylori* | T | 967 (5) | 53 | [17] |

## References


1 Leigh, G. Stock assessment of whaler and hammerhead sharks (Carcharhinidae and Sphyrnidae) in Queensland. 134 (2015).

2 Holmes, B. J. *et al.* Age and growth of the tiger shark Galeocerdo cuvier off the east coast of Australia. *Journal of Fish Biology* **87**, 422-448, doi:10.1111/jfb.12732 (2015).

3 Cortes, E. Life history patterns and correlations in sharks. *Reviews in Fisheries Science* **8**, 299-344 (2000).

4 Kleisner, K. *et al.* Australia: reconstructing estimates of total fisheries removals 1950-2010. 27 (2015).

5 Froese, R. & Pauly, D. *FishBase. World Wide Web electronic publication. www.fishbase.org, version (07/2016).* 2016).

6 Duffy, C. A. J., Francis, M. P., Manning, M. J. & Bonfil, R. in *Global Perspectives on the Biology and the Life History of the White Shark* (ed M. Domeier) Ch. 21, 301-318 (CRC Press, 2012).

7 Bruce, B. D., Stevens, J. D. & Malcolm, H. Movements and swimming behaviour of white sharks (Carcharodon carcharias) in Australian waters. *Marine Biology* **150**, 161-172, doi:10.1007/s00227-006-0325-1 (2006).

8 Bruce, B. D. & Bradford, R. W. Spatial dynamics and habitat preferences of juvenile white sharks : identifying critical habitat and options for monitoring recruitment : final report June 2008. 71 (CSIRO Marine and Atmospheric Research, 2008).

9 Heupel, M. R. *et al.* Conservation challenges of sharks with continental scale migrations. *Frontiers in Marine Science* **24**, 1-7 (2012).

10 Holmes, B. J. *et al.* Tiger shark (Galeocerdo cuvier) movement patterns and habitat use determined by satellite tagging in eastern Australian waters. *Marine Biology* **161**, 2645-2658, doi:10.1007/s00227-014-2536-1 (2014).

11 Werry, J. M. *et al.* Reef-fidelity and migration of tiger sharks, *Galeocerdo cuvier*, across the Coral Sea. *Plos One* **9**, e83249, doi:10.1371/journal.pone.0083249 (2014).

12 Ocearch. *http://www.ocearch.org/*, 2017).

13 Otway, N. M. & Ellis, M. T. Pop-up archival satellite tagging of Carcharias taurus: movements and depth/temperature-related use of south-eastern Australian waters. *Marine and Freshwater Research* **62**, 607-620, doi:10.1071/Mf10139 (2011).

14 Bansemer, C. S. & Bennett, M. B. Sex- and maturity-based differences in movement and migration patterns of grey nurse shark, Carcharias taurus, along the eastern coast of Australia. *Marine and Freshwater Research* **62**, 596-606, doi:10.1071/Mf10152 (2011).

15 Bruce, B. D., Stevens, J. D. & Bradford, R. W. Designing protected areas for grey nurse sharks off eastern Australia. 60 (CSIRO Marine and Atmospheric Research), 2005).

16 Otway, N. M. & Burke, A. L. Mark-recapture population estimate and movements of Grey Nurse Sharks. 63 (NSW Fisheries Conservation Research, 2004).

17 Harry, A. V. *et al.* Assessment of a data-limited, multi-species shark fishery in the Great Barrier Reef Marine Park and south-east Queensland. *Fisheries Research* **177**, 104-115, doi:10.1016/j.fishres.2015.12.008 (2016).

18 Heupel, M. R., Simpfendorfer, C. A. & Fitzpatrick, R. Large-Scale Movement and Reef Fidelity of Grey Reef Sharks. *Plos One* **5**, e9650, doi:10.1371/journal.pone.0009650 (2010).

19 Lough, J. M. Tropical river flow and rainfall reconstructions from coral luminescence: Great Barrier Reef, Australia. *Paleoceanography* **22**, Pa2218, doi:10.1029/2006pa001377 (2007).

20 Perry, A. L., Low, P. J., Ellis, J. R. & Reynolds, J. D. Climate change and distribution shifts in marine fishes. *Science* **308**, 1912-1915, doi:10.1126/science.1111322 (2005).

21 Schlaff, A. M., Heupel, M. R. & Simpfendorfer, C. A. Influence of environmental factors on shark and ray movement, behaviour and habitat use: a review. *Reviews in Fish Biology and Fisheries* **24**, 1089-1103, doi:10.1007/s11160-014-9364-8 (2014).

22 Heithaus, M. R. The biology of tiger sharks, Galeocerdo cuvier, in Shark Bay, Western Australia: Sex ratio, size distribution, diet, and seasonal changes in catch rates. *Environmental Biology of Fishes* **61**, 25-36, doi:Doi 10.1023/A:1011021210685 (2001).
23 Long, D. J. & Jones, R. E. in *Great white sharks: the biology of Carcharodon carcharias* (eds A.P. Klimley & D. G. Ainley) Ch. 27, 293-307 (Elsevier Academic Press, 1996).
24 Hammerschlag, N. *et al.* Behavioral evidence suggests facultative scavenging by a marine apex predator during a food pulse. *Behavioral Ecology and Sociobiology* **70**, 1777-1788, doi:10.1007/s00265-016-2183-2 (2016).
25 Department of Agriculture, F. a. F. *Safety at the beach: Queensland's shark safety program* <https://www.daf.qld.gov.au/__data/assets/pdf_file/0006/56823/Safety-at-the-beach-new-2012.pdf> (2012).
26 Guttridge, T. L., Myrberg, A. A., Porcher, I. F., Sims, D. W. & Krause, J. The role of learning in shark behaviour. *Fish and Fisheries* **10**, 450-469, doi:10.1111/j.1467-2979.2009.00339.x (2009).
27 Sumpton, W. D., Taylor, S. M., Gribble, N. A., McPherson, G. & Ham, T. Gear selectivity of large-mesh nets and drumlines used to catch sharks in the Queensland Shark Control Program. *African Journal of Marine Science* **33**, 37-43, doi:10.2989/1814232x.2011.572335 (2011).
28 Dudley, S. F. J., Haestier, R. C., Cox, K. R. & Murray, M. Shark control: experimental fishing with baited drumlines. *Marine and Freshwater Research* **49**, 653-661, doi:Doi 10.1071/Mf98026 (1998).
29 Patterson, R. Shark prevention measures working well. *Australian Fisheries*, 12-18 (1986).
30 Reid, D. D. & Krogh, M. Assessment of Catches from Protective Shark Meshing Off New-South-Wales Beaches between 1950 and 1990. *Australian Journal of Marine and Freshwater Research* **43**, 283-296 (1992).
31 Dudley, S. F. J. & Cliff, G. Some effects of shark nets in the Natal nearshore environment. *Environmental Biology of Fishes* **36**, 243-255 (1993).
32 Patterson, R. A. Effects of long-term anti-shark measures on target and non-target species in Queensland, Australia. *Biological Conservation* **52**, 147-159 (1990).
33 Rako, N. *et al.* Leisure boating noise as a trigger for the displacement of the bottlenose dolphins of the Cres-Losinj archipelago (northern Adriatic Sea, Croatia). *Marine Pollution Bulletin* **68**, 77-84, doi:10.1016/j.marpolbul.2012.12.019 (2013).
34 Morton, A. B. & Symonds, H. K. Displacement of Orcinus orca (L.) by high amplitude sound in British Columbia, Canada. *Ices Journal of Marine Science* **59**, 71-80, doi:DOI 10.1006/jmsc.2001.1136 (2002).
35 Filous, A. *et al.* Displacement effects of heavy human use on coral reef predators within the Molokini Marine Life Conservation District. *Marine Pollution Bulletin* **121**, 274-281, doi:10.1016/j.marpolbul.2017.06.032 (2017).
36 Coppleson, V. & Goadby, P. *Shark Attack.* (Angus & Robertson, 1988).
37 Hazin, F. H. V., Afonso, A. S., De Castilho, P. C., Ferreira, L. C. & Rocha, B. C. L. M. Regional movements of the tiger shark, Galeocerdo cuvier, off northeastern Brazil: inferences regarding shark attack hazard. *Anais Da Academia Brasileira De Ciencias* **85**, 1053-1062, doi:Doi 10.1590/S0001-37652013005000055 (2013).
38 Hazin, F. H. V., Burgess, G. H. & Carvalho, F. C. A shark attack outbreak off Recife, Pernambuco, Brazil: 1992-2006. *Bulletin of Marine Science* **82**, 199-212 (2008).
39 Brunnschweiler, J. M. & Baensch, H. Seasonal and Long-Term Changes in Relative Abundance of Bull Sharks from a Tourist Shark Feeding Site in Fiji. *Plos One* **6**, doi:10.1371/journal.pone.0016597 (2011).

40 Maljkovic, A. & Cote, I. M. Effects of tourism-related provisioning on the trophic signatures and movement patterns of an apex predator, the Caribbean reef shark. *Biological Conservation* **144**, 859-865, doi:10.1016/j.biocon.2010.11.019 (2011).
41 Bruce, B. D. & Bradford, R. W. The effects of shark cage-diving operations on the behaviour and movements of white sharks, Carcharodon carcharias, at the Neptune Islands, South Australia. *Marine Biology* **160**, 889-907, doi:10.1007/s00227-012-2142-z (2013).
42 Bohnet, I. C. & Pert, P. L. Patterns, drivers and impacts of urban growth-A study from Cairns, Queensland, Australia from 1952 to 2031. *Landscape and Urban Planning* **97**, 239-248, doi:10.1016/j.landurbplan.2010.06.007 (2010).
43 Spearritt, P. The 200 Km City: Brisbane, the Gold Coast, and Sunshine Coast. *Aust Econ Hist Rev* **49**, 87-106, doi:10.1111/j.1467-8446.2009.00251.x (2009).
44 Mullins, P. Tourist cities as new cities: Australia's Gold Coast and Sunshine Coast. *Australian Planner* **28**, 37-41 (1990).
45 Chilvers, B. L. *et al.* Moreton Bay, Queensland, Australia: an example of the co-existence of significant marine mammal populations and large-scale coastal development. *Biological Conservation* **122**, 559-571, doi:10.1016/j.biocon.2004.08.013 (2005).
46 Gribble, N., Whybird, O., Williams, L. & Garrett, R. Fishery assessment update 1988-2003: Queensland East Coast shark. 26 (DPI & F Animal Science, 2005).
47 GBRMPA. Great Barrier Reef Marine Park Authority Position Statement on the conservation and management of sharks and rays in the Queensland East Coast Inshore Finfish Fishery. (2007).
48 Harry, A. V. *et al.* Evaluating catch and mitigating risk in a multispecies, tropical, inshore shark fishery within the Great Barrier Reef World Heritage Area. *Marine and Freshwater Research* **62**, 710-721, doi:10.1071/Mf10155 (2011).
49 Rose, C. & McLoughlin, K. Review of shark finning in Australian fisheries : final report to Fisheries Resources Research Fund. 172 (Bureau of Rural Sciences, Canberra, 2001).
50 Koopman, M. & Knuckey, I. Advice on CITES Appendix II Shark Listings. Report to Department of Sustainability, Environment, Water, Population and Communities. 144 (Fishwell Consulting, 2014).
51 Pears, R. J. *et al.* East Coast Otter Trawl Fishery in the Great Barrier Reef Marine Park: technical report. 276 (Great Barrier Reef Marine Park Authority, Townsville, 2012).
52 AFMA. Ecological risk management report for the eastern tuna and billfish fishery. 15 (2012).
53 Stevens, J. D. & Wayte, S. E. A review of Australia's pelagic shark resources. 70 (2012).
54 Government, Q. Report on the Bycatch and byproduct risk assessment for the East Coast Spanish Mackerel Fishery. 49 (Department of Primary Industries and Fisheries, 2005).
55 Macbeth, W. G., Geraghty, P. T., Peddemors, V. M. & Gray, C. A. Observer-based Study of Targeted Commercial Fishing for Large Shark Species in Waters off Northern New South Wales. . 82 (2009).
56 Worm, B. *et al.* Global catches, exploitation rates, and rebuilding options for sharks. *Marine Policy* **40**, 194-204, doi:10.1016/j.marpol.2012.12.034 (2013).
57 Industries, N. D. o. P. Ocean Trap and Line Fisheries Environmental Impact Statement 177 (NSW Department of Primary Industries, Cronulla, 2006).
58 Juncker, M. & Clua, E. Coastal shark fisheries in the pacific: A brief overview of current knowledge. 27 (2006).
59 Tull, M. in *Historical perspectives of fisheries exploitation in the Indo-Pacific* (eds J. Christensen & M. Tull) 63-83 (Springer 2014).
60 Skewes, T. D. Marine Resource Profiles: Solomon Islands. 63 (Forum Fisheries Agency, 1990).
61 Kumoro, L. The shark longline fishery in Papua New Guinea 5(2003).

62 de Faria, F. *Recreational fishing of sharks in the Great Barrier Reef World Heritage Area: species composition and incidental capture stress* MSc thesis, James Cook University (2012).
63 Gallagher, A. J., Hammerschlag, N., Danylchuk, A. J. & Cooke, S. J. Shark recreational fisheries: Status, challenges, and research needs. *Ambio* **46**, 385-398, doi:10.1007/s13280-016-0856-8 (2017).
64 Chin, A. in *State of the Great Barrier Reef On-line* (ed A. Chin) (Great Barrier Reef Marine Park Authority, 2005).
65 Stevens, J. D. Biological Observations on Sharks Caught by Sport Fishermen Off New-South-Wales. *Australian Journal of Marine and Freshwater Research* **35**, 573-590 (1984).
66 Murphy, J. J., Lowry, M. B., Henry, G. W. & Chapman, D. Gamefishing tournament monitoring program-1993 to 2000. *NSW Fisheries Final Report Series*, 38 (2002).
67 Green, A. L. *et al.* Larval dispersal and movement patterns of coral reef fishes, and implications for marine reserve network design. *Biological Reviews* **90**, 1215-1247, doi:10.1111/brv.12155 (2015).
